# Pro-angiogenic Activity Discriminates Human Adipose-Derived Stromal Cells From Retinal Pericytes: Considerations for Cell-Based Therapy of Diabetic Retinopathy

**DOI:** 10.3389/fcell.2020.00387

**Published:** 2020-06-09

**Authors:** Heiner Kremer, Julian Gebauer, Susanne Elvers-Hornung, Stefanie Uhlig, Hans-Peter Hammes, Elena Beltramo, Lothar Steeb, Martin C. Harmsen, Carsten Sticht, Harald Klueter, Karen Bieback, Agnese Fiori

**Affiliations:** ^1^Institute of Transfusion Medicine and Immunology, Medical Faculty Mannheim, Heidelberg University, Mannheim, Germany; ^2^German Red Cross Blood Donation Service Baden-Württemberg – Hessen, Mannheim, Germany; ^3^FlowCore Mannheim, Medical Faculty Mannheim, Heidelberg University, Mannheim, Germany; ^4^5th Medical Department, Medical Faculty Mannheim, Heidelberg University, Mannheim, Germany; ^5^Department of Medical Sciences, University of Turin, Turin, Italy; ^6^PeloBiotech GmbH, Martinsried, Germany; ^7^Department of Pathology and Medical Biology, University Medical Center Groningen, University of Groningen, Groningen, Netherlands; ^8^Center for Medical Research, Medical Faculty Mannheim, Heidelberg University, Mannheim, Germany; ^9^Mannheim Institute for Innate Immunoscience, Medical Faculty Mannheim, Heidelberg University, Mannheim, Germany; ^10^HEiKA—Heidelberg Karlsruhe Strategic Partnership, Karlsruhe Institute of Technology (KIT), Heidelberg University, Heidelberg, Germany

**Keywords:** human adipose-derived stromal cells, human retinal pericytes, diabetic retinopathy, angiogenesis, vascular–endothelial growth factor, angiopoietin

## Abstract

Diabetic retinopathy (DR) is a frequent diabetes-associated complication. Pericyte dropout can cause increased vascular permeability and contribute to vascular occlusion. Adipose-derived stromal cells (ASC) have been suggested to replace pericytes and restore microvascular support as potential therapy of DR. In models of DR, ASC not only generated a cytoprotective and reparative environment by the secretion of trophic factors but also engrafted and integrated into the retina in a pericyte-like fashion. The aim of this study was to compare the pro-angiogenic features of human ASC and human retinal microvascular pericytes (HRMVPC) *in vitro*. The proliferation and the expression of ASC and HRMVPC markers were compared. Adhesion to high glucose-conditioned endothelial extracellular matrix, mimicking the diabetic microenvironment, was measured. The angiogenesis-promoting features of both cell types and their conditioned media on human retinal endothelial cells (EC) were assessed. To identify a molecular basis for the observed differences, gene expression profiling was performed using whole-genome microarrays, and data were validated using PCR arrays and flow cytometry. Based on multiplex cytokine results, functional studies on selected growth factors were performed to assess their role in angiogenic support. Despite a distinct heterogeneity in ASC and HRMVPC cultures with an overlap of expressed markers, ASC differed functionally from HRMVPC. Most importantly, the pro-angiogenic activity was solely featured by ASC, whereas HRMVPC actively suppressed vascular network formation. HRMVPC, in contrast to ASC, showed impaired adhesion and proliferation on the high glucose-conditioned endothelial extracellular matrix. These data were supported by gene expression profiles with differentially expressed genes. The vessel-stabilizing factors were more highly expressed in HRMVPC, and the angiogenesis-promoting factors were more highly expressed in ASC. The vascular endothelial growth factor receptor-2 inhibition efficiently abolished the ASC angiogenic supportive capacities, whereas the addition of angiopoietin-1 and angiopoietin-2 did not alter these effects. Our results clearly show that ASC are pro-angiogenic, whereas HRMVPC are marked by anti-angiogenic/EC-stabilizing features. These data support ASC as pericyte replacement in DR but also suggest a careful risk-to-benefit analysis to take full advantage of the ASC therapeutic features.

## Introduction

Diabetic retinopathy (DR) is a common microvascular complication of diabetes mellitus with a risk of causing blindness (Stitt et al., 2016; Hammes, 2018). The early stage of the disease, known as non-proliferative DR, is diagnosed by microvascular abnormalities. These are the consequence of a sequela of detrimental events, which involve the whole neurovascular retina and include pericyte dropout, basal lamina thickening, and endothelial, neuronal, and glial dysfunction (Stitt et al., 2016; Fiori et al., 2018; Hammes, 2018). Reactive, uncontrolled mechanisms cause angiogenesis, leading to proliferative DR. To date, treatments of sight-threatening DR include laser photocoagulation, vitreoretinal surgery, corticosteroids, and—more recently—anti-vascular endothelial growth factor (VEGF) drugs to limit and reduce the pathological hyperproliferation of retinal vessels (Stitt et al., 2016; Hammes, 2018). To avoid the associated side effects, cell-based therapies have been suggested (Stitt et al., 2011).

Loss of pericytes appears as the earliest event, causing destabilization of the retinal vessels (Pfister et al., 2013). Destructive signaling pathways involving the angiopoietin–Tie2 axis can lead to pericyte detachment and migration and, sometimes, subsequent apoptosis (Beltramo and Porta, 2013). Therefore, the prevention and/or the containment of DR progression may involve the control of pericyte dropout to promote vascular repair and the reversal of ischemic injury.

Therapy with mesenchymal stromal cells (MSC), especially those sourced from adipose tissue (adipose-derived stromal cells, ASC), emerged as an interesting treatment option for DR due to their pro-regenerative, pro-angiogenic, anti-apoptotic, and anti-inflammatory functions and their close relationship to pericytes (Fiori et al., 2018). Indeed the first hint came from a study where intravenously infused MSC improved the blood–retina barrier integrity (Yang et al., 2010). At this stage, it was not clear whether the observed effect was directly linked to the local action of the infused cells or secondary to lowered hyperglycemia. Subsequent data provided better insight, indicating that ASC not only generate a cytoprotective and regenerative environment by secretion of trophic factors acting on endothelial, neuronal, and glial cells (Ezquer et al., 2016) but also engraft and integrate into the retina in a pericyte-like fashion (Mendel et al., 2013). In different models of DR, ASC wrapped around retinal vessels and expressed α smooth muscle actin (α-SMA) upon intravitreal injection. This went with an improvement of visual function and delay in disease progression (Mendel et al., 2013; Rajashekhar et al., 2014; Hajmousa et al., 2018). *In vitro* tube formation assays complemented these observations, indicating that ASC can support and stabilize capillary structures (Merfeld-Clauss et al., 2010). However, there are discrepant data on whether ASC can effectively migrate, integrate, and differentiate gaining pericyte-like functions or rather exert their function by paracrine effects. Ezquer et al. (2016) observed that the cells remained in the vitreous without signs of differentiation and acted *via* secreted factors. In contrast, (Cronk et al., 2015) observed that only cells, but not the conditioned medium, were vasoprotective. Our previous data indicate that cell–cell interactions *via* NOTCH-2 are required for *in vitro* tube formation, but not for *in vivo* angiogenesis, which appeared to be independent of NOTCH-2, mainly based on paracrine factors (Terlizzi et al., 2018).

Besides the uncertainties in understanding the effective mode of action of MSC/ASC in DR, another concern regarding MSC therapy is that the cells should resist the diabetic microenvironment, which may impair the MSC pro-regenerative function (Cianfarani et al., 2013; Rennert et al., 2014; Cronk et al., 2015). However, others and we have shown that ASC resist hyperglycemic stress and restore the angiogenic properties of endothelial cells (EC) which were suppressed by hyperglycemia (Rajashekhar et al., 2014; Hajmousa et al., 2018; Fiori et al., 2020). Furthermore, the MSC-mediated secretion of pro-angiogenic factors may represent a significant risk as these factors may worsen DR by promoting vessel proliferation (Beltramo et al., 2014). In fact, we observed that the intravitreal injection of MSC into non-diabetic transgenic animals induced cataract, pericyte loss, vascular dysfunction, and inflammatory responses, thus worsening the established retinopathy (Huang et al., 2019).

To clarify whether ASC can take over pericyte functions, we compared the pro-angiogenic and the pericyte-like functions of human ASC and human retinal microvascular pericytes (HRMVPC) *in vitro* to elucidate the differences and the similarities between these two cell types. Intriguingly, the pro-angiogenic activity was solely featured by ASC, whereas HRMVPC actively suppressed the vascular network formation.

## Materials and Methods

### Cell Culture

All protocols for isolating the primary cells were approved by the Mannheim Ethics Commission II, except for the cells provided by collaboration or commercial sources. All donors gave written informed consent in accordance with the Declaration of Helsinki.

Human umbilical vein endothelial cells (HUVEC), initially used as a model of EC, were isolated as described before (Bieback et al., 2013) and cultivated at 8,000 cells/cm^2^ in endothelial cell growth medium-2 (ECGM-2 with 1 g/L glucose; PromoCell, Heidelberg, Germany). HUVEC, derived from different donors, were used from passages 3 to 5. Human retinal microvascular endothelial cells (HRMVEC; two different donor isolates, passage 3; PeloBiotech, Planegg, Germany), used to reflect the microvascular retinal milieu, were cultured similar to HUVEC and used until passage 8. For some experiments, EC were cultured in normal glucose (NG; 1 g/L standard concentration in all culture media used), high glucose (HG; 4.5 g/L), or mannitol as osmotic control, adding additional 3.5 g/L glucose/mannitol to the media. Adipose-derived stromal cells (ASC) were isolated from lipoaspirate as described previously (Bieback et al., 2012). The ASC were cultured at seeding densities of 200 ASC/cm^2^ in Dulbecco’s modified Eagles medium (DMEM) with 10% human AB serum (1 g/L glucose; PAN Biotech, Aidenbach, Germany and German Red Cross Blood Donor Service, respectively), 1% penicillin/streptomycin (PAN Biotech), and 2% L-glutamine (200 mM; PAN Biotech). Different donor isolates, passages 2 to 4, were used throughout the study to account for donor-specific variances. The immortalized human retinal pericytes (Bmi-HRMVPC; passage 6; kindly provided by Elena Beltramo), initially used for comparison, were cultured by seeding 20,000 cells/cm^2^ in DMEM 10% fetal bovine serum (FBS; Sigma-Aldrich Chemie GmbH, Munich, Germany) and used at passages 7 to 8. In addition, three different isolates of primary human retinal microvascular pericytes (HRMVPC, passage 3) were purchased to account for donor-specific differences. The HRMVPC were cultured by seeding 20,000 cells/cm^2^ in pericyte growth medium in flasks coated with Speed Coating Solution (all PeloBiotech) and used from passages 4 to 6.

All cells were cultured at 37°C with 5% CO_2_ and the medium was changed every 2 to 3 days. All cells were cryopreserved using 90% FBS and 10% dimethyl sulfoxide (Wak-chemie Medical GmbH, Steinbach, Germany).

#### GFP and dTomato Expressing HUVEC and HRMVEC

To monitor angiogenesis, EC were transduced to express GFP or dTomato. The GFP- or dTomato-expressing plasmids pHR’SIN-cPPT-SEW, together with pCMV-DR8.91 and pMD.G (all kindly provided by Prof. Patrick Maier, Department of Radiation Oncology, University Medical Centre Mannheim, Germany), were used to produce lentiviral vectors through the transient transfection of 293FT cells. The HUVEC and HRMVEC were transduced once in the presence of polybrene (8 μg/ml; Sigma-Aldrich). The transduced GFP- or dTomato-positive EC were sorted using a BD FACSAria IIu (Becton Dickinson, Heidelberg, Germany), collected, and cultured as reported previously. The transduced EC were used from passages 5 to 11.

### Comparative Characterization of Human ASC and HRMVPC

The growth curves of ASC, HRMVPC, and Bmi-HRMVPC were assessed by seeding 2,500 cells/cm^2^ in 96-well plates in eight technical replicates and recording the increasing confluence every 2 to 4 h using a live imaging device (IncuCyte ZOOM; Essen BioScience, Ann Arbor, MI, United States) (Supplementary Figure S1). The percent confluence was determined using an adapted processing definition (IncuCyte^®^ ZOOM software).

Multiparametric flow cytometry was performed on 10,000 trypsinized cells using the titrated antibodies listed in Supplementary Table S1, using a BD FACSCanto II (Becton Dickinson) running BD FACSDIVA software. The obtained data were analyzed with FlowJo software (FlowJo, LLC, Ashland, OR, United States). The percentage positivity and the mean fluorescence intensity (MFI) values were calculated against unstained controls. All antibodies were validated using a positive control, except for the regulator of G protein signaling 5 (RGS5), where putative positive controls yielded negative results despite using different antibody clones and direct, indirect, extracellular, and intracellular staining. These data, thus, are not shown.

### Interaction of ASC and HRMVPC With High Glucose-Conditioned Endothelial Extracellular Matrix

To compare the interaction between ASC, Bmi-HRMVPC, and HRMVPC on HG-conditioned EC extracellular matrix (ECM), we followed the protocol by Beltramo et al. (2009) with slight modifications. Briefly, the HUVEC (15,000 cells/cm^2^) or the HRMVEC (7,500 or 10,000 cells/cm^2^) were cultured in 24-well plates. The HUVEC were seeded in ¼ ECGM-2 (ECGM-2 with 75% reduced growth supplements) at NG or HG conditions. We have previously observed that only upon lowering the growth factor content did the negative HG effects on EC became apparent (Fiori et al., 2020). Mannitol (3.5 g/L) served as osmotic control. The HRMVEC were seeded in standard ECGM-2 at NG or HG conditions. Upon reaching at least 95% confluence, as monitored by kinetic live cell imaging (IncuCyte ZOOM), the EC were washed once with phosphate-buffered saline (PBS) and lysed with 0.25 mM ammonia solution for 3 min. The obtained ECM was washed thrice with PBS and kept wet until use. The intactness of the ECM was verified by brilliant blue stain (Supplementary Figure S2). The ASC or HRMVPC were seeded on top of the ECM at a density of 5,000 cells/cm^2^. Using kinetic live imaging, adhesion was monitored after 10 and 20 min and proliferation was monitored every 2 h for 2 days. The average single cell area was assessed during adhesion and cell confluence/proliferation using individually adapted segmentation masks/processing definitions (Supplementary Figure S2). Single measurements were excluded when confounded by debris or image analysis errors. The average of the technical replicate values in each individual experiment was normalized to NG control and used to calculate the mean values and the standard deviation for the biological replicates.

### Angiogenesis Assays

To assess the vascular network/tube formation of EC and the pro-angiogenic potential of ASC and HRMVPC, two angiogenesis assays were used—one dedicated to assess the supportive action of the ASC/HRMVPC seeded as a monolayer and the other to assess the pro-angiogenic activity of the conditioned medium (CM).

#### Coculture (CC) Angiogenesis Assay

A total of 30,000 ASC or HRMVPC were seeded per well in a 96-well plate in ¼ ECGM-2 for 1 h, and ¼ ECGM-2 was used to keep the endothelial growth factor concentrations low. Then, 5,000 (in later passages 8,000) GFP or dTomato HUVEC or GFP or dTomato HRMVEC were seeded in ¼ ECGM-2 on top of the monolayer. The cocultures were incubated for 72 h. Visualization of tube formation over time was performed by taking phase-contrast and fluorescent images with the IncuCyte ZOOM imaging device (Supplementary Figure S3A). An integrated angiogenesis algorithm measured tube formation by calculating the tube length, area, and number of branch points. After 72 h of coculture, CM was collected and frozen at −80°C. The respective monoculture-derived CM was prepared in parallel; ¼ ECGM-2 served as control.

#### Basal Lamina Matrix (BM) Angiogenesis Assay

A total of 17,000 GFP or dTomato HRMVEC were seeded per well in a 96-well plate on top of a 50-μl/well layer of a basal lamina matrix (Geltrex^TM^ LDEV-free reduced growth factor matrix; Thermo Fisher Scientific, United States) in ¼ ECGM-2 or CM of the CC angiogenesis assay. Fluorescent images were taken every 30 min with the IncuCyte ZOOM live imaging device (Supplementary Figure S3A). Network formation was monitored for up to 6 h and maximum values of branch points (1/mm^2^) and network length (mm/mm^2^) were calculated using either NIH ImageJ with the Angiogenesis Analyzer plugin or the IncuCyte Software. Network branch point metrics were used for statistical analyses. Because ASC showed network formation in the BM angiogenesis assay similar to EC, we did not run direct cocultures in this assay but used it to analyze the CM effects on HRMVEC tube formation.

#### Conditioned Medium, Inhibitors, and Growth Factor Addition

When specified, CM derived from either CC angiogenesis assays or monocultures, cultured for 72 h in ¼ ECGM-2, was added to both angiogenesis assays instead of the control medium. Furthermore, the following substances were used: recombinant human vascular endothelial growth factor (rhVEGF_165_, 10 ng/ml; PeproTech, London, United Kingdom), angiopoietin-1 and angiopoietin-2 [rhAng-1 and rhAng-2, 400 ng/ml (Teichert et al., 2017); both PeproTech], suramin sodium (anti-angiogenic compound, 100 μM; Santa Cruz Biotechnology, Heidelberg, Germany), and ZM 323881 hydrochloride [selective VEGFR2 antagonist, 1 μM (Busceti et al., 2017); Santa Cruz Biotechnology].

#### Immunofluorescence Staining

To assess pericyte-like differentiation upon network formation, the expression of pericyte markers α-smooth muscle actin (α-SMA), neural/glial antigen 2 (NG-2), regulator of G protein signaling 5 (RGS5), and platelet-derived growth factor receptor-β (PDGFR-β) (Bergers and Song, 2005) was assessed in CC angiogenesis cocultures. A total of 100,000 ASC/well were seeded in ¼ ECGM-2 medium in eight-well μ-slides (ibidi, Gräfelfing, Germany) and then 20,000 dTomato HRMVEC were seeded on top. Network formation was monitored over 72 h. Then, the wells were washed with PBS and fixed with 2% paraformaldehyde in PBS for 30 min. After washing, the cells were permeabilized with 0.5% Triton X-100. After 15 min of blocking with 2% bovine serum albumin (BSA), 50 μl/well of ready-to-use mouse anti-human α-SMA (Progen, Heidelberg, Germany), mouse anti-human NG-2 (1:100; Santa Cruz Biotechnology), mouse anti-human RGS5 (RGS5 and RGS5-AF647 clone B4, 1:100; Santa Cruz Biotechnology or clone OTI1C1; OriGene Technologies, Rockville, MD, United States), or mouse anti-human PDGFR-β (clone 18A2, 1:100; Santa Cruz Biotechnology) were added and incubated for 1 h in the dark. After washing with 0.1% BSA, secondary antibody anti-mouse-Alexa Fluor 488 F(ab’)2 (1:1,000 final dilution, Invitrogen, Thermo Fisher Scientific, Karlsruhe, Germany) was added for 1 h in the dark. The nuclei were counterstained with DAPI (1 μg/ml; Sigma-Aldrich) before embedding the slides in the mounting medium (ibidi). The images were captured using either a Zeiss microscope with AxioVision Rel. 4.7 (Carl Zeiss Microscopy GmbH, Jena, Germany) or an inverse Leica SP5 Mid-multi-photon system (Leica, Wetzlar, Germany) using a × 25 (0.95 NA) water immersion objective or × 40/1.3 NA oil objective (Leica). The negative controls without first antibody showed no staining. RGS5 staining gave negative results and, because antibodies could not be validated using a positive control, the results are not shown.

### Microarray and PCR Array Validation

#### RNA Isolation

The ASC and HRMVPC (three biological replicates each) were cultured for 7 days. Then, the cells were trypsinized, washed, and RNA-isolated using Qiagen RNeasy mini Kit (Qiagen, Hilden, Germany). RNA quality was tested by capillary electrophoresis on an Agilent 2100 bioanalyzer (Agilent, Santa Clara, CA, United States) and high quality (RNA integrity values ≥9) was confirmed.

#### Microarray

Gene expression profiling was performed using human HTA-2_0-st-type arrays (Affymetrix, Thermo Fisher Scientific). The biotinylated antisense cRNA was prepared, according to the standard labeling protocol, with the GeneChip^®^ WT Plus Reagent Kit and the GeneChip^®^ Hybridization, Wash, and Stain Kit. The hybridization on the chip was performed on a GeneChip Hybridization oven 640, then dyed in the GeneChip Fluidics Station 450, and thereafter scanned with a GeneChip Scanner 3000.

#### Bioinformatics

A custom CDF version 22 with ENTREZ-based gene definitions was used to annotate the arrays (Dai et al., 2005). The raw fluorescence intensity values were normalized by applying quantile normalization and RMA background correction. An ANOVA was performed to identify differentially expressed genes using a commercial software package (SAS JMP11 Genomics, version 7; SAS Institute, Cary, NC, United States). A false positive rate of a = 0.05 with FDR correction was taken as the level of significance. Gene Set Enrichment Analysis (GSEA) was used to determine whether the defined sets of genes exhibit a statistically significant bias in their distribution within a ranked gene list using the R software packages EnrichmentBrowser (Geistlinger et al., 2016). KEGG pathway analysis was performed, focusing on apparently relevant pathways^1^.

The raw and normalized data are deposited in the Gene Expression Omnibus database (accession no. GSE144605)^2^.

#### PCR Array

The selected genes were validated using RT2 Custom Profiler PCR Arrays (Supplementary Table S2; Qiagen, Hilden, Germany), according to the manufacturer’s instructions, using a LightCycler^®^ 480 (Roche Life Science, Mannheim, Germany). These PCR arrays were also performed on RNA samples from HUVEC and HRMVEC cultured for 5 days in ¼ ECGM-2 NG/HG (three and one biological replicate, respectively).

### Multiplex Cytokine Analysis of Conditioned Media

The angiogenic growth factors in CM derived from either CC angiogenesis assays or monocultures were analyzed with a bead-based immunoassay (LEGENDplex^TM^ Human Angiogenesis Panel 1; BioLegend, San Diego, CA, United States) by following the manufacturer’s instructions. Briefly, the samples and the standards were loaded in a 96-well V-bottom plate, followed by incubation with premixed capture beads for 2 h at room temperature (RT) on a plate shaker. After two washing steps with wash buffer, the detection antibodies were added for 1 h at RT to form capture bead/analyte/detection antibody sandwiches. LEGENDplex^TM^ Streptavidin-PE was then added directly after incubation for 30 min at RT. After a final washing step, the samples were transferred to fluorescence-activated cell sorting tubes and analyzed using BD FACS Canto II (Becton Dickinson). The data were analyzed with the LEGENDplex^TM^ data analysis software (BioLegend).

### Statistical Analysis

All results are expressed as mean ± standard deviation; *n* represents the number of biological replicates or, if not possible to test the different donors, the technical replicates in independent experiments. Statistical analyses were performed using GraphPad Prism 7 (GraphPad Software, San Diego, CA, United States). Differences between experimental groups were analyzed after normality testing by one- or two-way ANOVA (repeated-measures, RM, if applicable) with *post hoc* tests as indicated. *P* ≤ 0.05 was considered as statistically significant (^∗^*p* ≤ 0.05, ^∗∗^*p* ≤ 0.01, ^∗∗∗^*p* ≤ 0.001, and ^****^p ≤ 0.0001). For comparison and correlation analysis of the microarray, PCR array, and flow cytometry data, the programming language R was used. Volcano plots were generated using the R package ggplot2.

## Results

### ASC and Retinal Pericyte Morphology and Growth Curves Differ, but the MSC Marker Expression Is Similar

**FIGURE 1:**
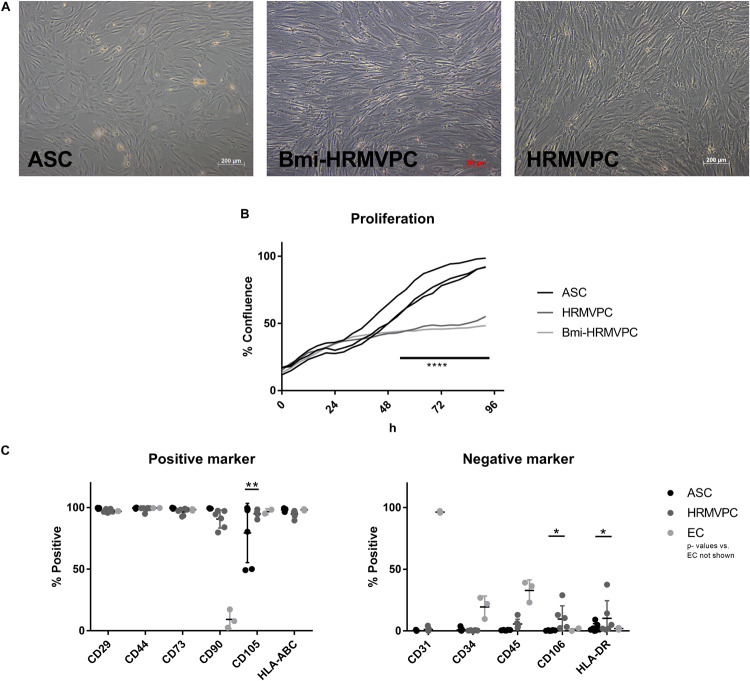
Morphology of adipose-derived stromal cells (ASC) and human retinal microvascular pericytes (HRMVPC) and the growth curves differ, whereas the mesenchymal stromal cells (MSC) marker expression is indistinguishable. **(A)** Representative images of ASC, Bmi-HRMVPC and HRMVPC. **(B)** Growth curves of ASC (three different donors), HRMVPC (one donor), and Bmi-HRMVPC cell line determined by kinetic live imaging (****p ≤ 0.0001, two-way ANOVA with Sidak’s *post hoc* test). **(C)** Surface expression of MSC positive and negative markers for ASC (*n* = 7 different donors), HRMVPC (HRMVPC and Bmi-HRMVPC groups, *n* = 6, biological and technical replicates from three different donors), and endothelial cells (EC; *n* = 3, human umbilical vein endothelial cell and human retinal microvascular endothelial cell groups) (*p ≤ 0.05 and **p ≤ 0.01, two-way ANOVA with Tukey’s *post hoc* test; significant differences to EC not depicted).

To compare the basic characteristics of ASC and HRMVPC, cell morphology, growth curves, and expression of typical MSC markers were assessed. Both cell types had a typical fibroblastoid morphology, but both immortalized Bmi-HRMVPC, used first for comparison, and HRMVPC, used later to account for potential donor differences, appeared more elongated and slender with an apparently higher light diffraction at the cell borders (Figure 1A). Both HRMVPC detached within seconds after adding trypsin, in contrast to ASC that needed approximately 5 min to detach. This suggests that different proteins are involved in cell adhesion, substantiated by the recommended coating for primary HRMVPC. The cell sizes were comparable after trypsinization and ranged between 19 and 21 μm. Both HRMVPC required high seeding densities (split of maximum 1:5), whereas ASC could be seeded at low densities (200 cells/cm^2^) to obtain optimal cell proliferation. This also suggests different needs for cell–cell contacts for proliferation. Using live cell imaging, a direct comparison of the growth curves was performed by seeding cells at 2,500 cells/cm^2^ and assessing the increase of confluence (Supplementary Figure S1). For the first 24 h, the confluence increased indistinguishably, indicating similar kinetics for cell adhesion and spreading (Figure 1B). Thereafter, HRMVPC proliferation was very slow in contrast to ASC, which showed a sigmoid curve at reaching 100% confluence after 96 h.

The ASC are characterized by the expression/non-expression of certain surface markers (Dominici et al., 2006; Bourin et al., 2013). Flow cytometry analyses showed that marker expression was largely identical for ASC and HRMVPC (Figure 1C). The heterogeneity in endoglin (CD105) expression—two isolates with only 50% of the cells being positive—was surprising for ASC since in previous studies all ASC uniformly expressed CD105 (Kern et al., 2006). The HRMVPC were more heterogeneous than ASC for Thy-1 (CD90, mean 90.7 ± 7.3%, *p* ≤ 0.01), CD45 (mean 5.6 ± 3.9%, non-significant), vascular cell adhesion molecule-1 (VCAM-1, CD106, mean: 9.7 ± 10.7%, *p* ≤ 0.05), and HLA-DR (mean 10.3 ± 14.3%, *p* ≤ 0.05). The EC displayed a significantly differing phenotype as expected (*p* value not shown).

### HRMVPC Show Impaired Interaction With HG-Conditioned EC ECM Compared to ASC

**FIGURE 2:**
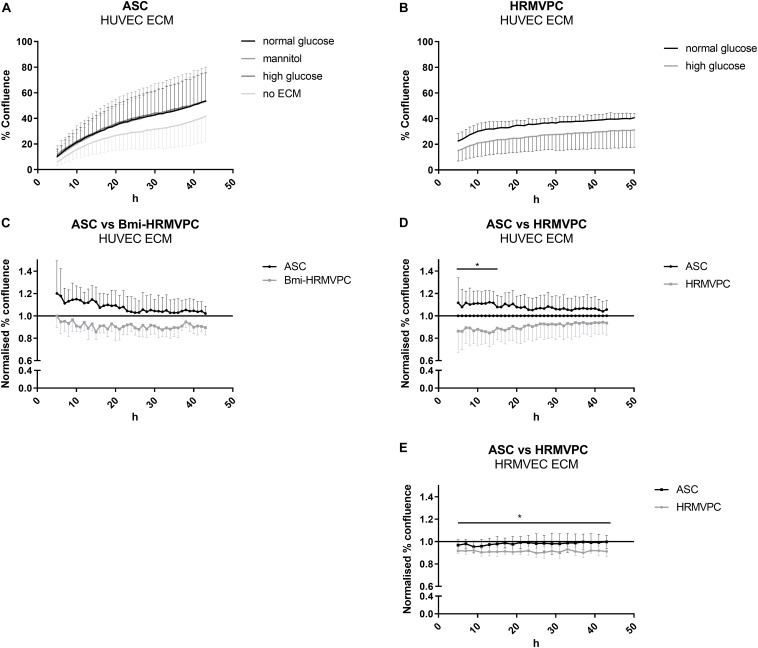
Human retinal microvascular pericytes (HRMVPC) show impaired interaction with high glucose (HG)-conditioned endothelial cells (EC) extracellular matrix (ECM) compared to adipose-derived stromal cells (ASC). The EC, either human umbilical vein endothelial cells (HUVEC) or human retinal microvascular endothelial cells (HRMVEC), were grown until confluence in normal glucose (NG), HG, or mannitol-supplemented medium. Then, the EC ECM was prepared as described in section “Materials and Methods”. ASC or HRMVPC were seeded on top, and the increase in confluence was monitored using live cell imaging over 48 h. **(A)** Confluence of ASC seeded on HUVEC ECM conditioned by NG, HG, or mannitol (*n* = 4 biological replicates; non-significant, two-way RM-ANOVA with Dunnett’s *post hoc* test). **(B)** Confluence of HRMVPC seeded on HG-conditioned HRMVEC ECM compared to NG-conditioned ECM (*n* = 5 independent experiments with HRMVPC from one donor; non-significant, two-way RM-ANOVA with Sidak’s *post hoc* test). **(C)** To account for differences in proliferative capacity, confluence data were normalized against the NG-conditioned HUVEC ECM (line at value 1). ASC vs. Bmi-HRMVPC (*n* = 3 independent experiments with ASC from different donors; non-significant, two-way RM-ANOVA with Sidak’s *post hoc* test). **(D)** ASC vs. HRMVPC interaction with NG and HG-conditioned HUVEC ECM (*n* = 5 independent experiments with ASC from different donors; *p ≤ 0.05 for ASC vs. HRMVPC for the first 14 h, two-way RM-ANOVA with Sidak’s *post hoc* test). **(E)** ASC vs. HRMPVC interaction with NG and HG-conditioned HRMVEC ECM (*n* = 3 independent experiments with ASC from different donors; *p ≤ 0.05 for HRMVPC interaction with NG and HG-conditioned HRMVEC ECM for all time points, two-way RM-ANOVA with Sidak’s *post hoc* test).

Beltramo et al. (2009) have shown that HG-conditioned EC ECM reduces the adhesion and increases the apoptosis of pericytes, mimicking the pericyte dropout found in DR. Hypothesizing that ASC can resist these changes, adhere, and proliferate, we compared the interaction of ASC and HRMVPC with NG- and HG-conditioned EC ECM. First, we tested HUVEC and then—to better mimic the microvasculature of the retina—HRMVEC. Furthermore, we assessed first immortalized Bmi-HRMVPC and later confirmed these data with primary HRMVPC from different donors. Using live cell imaging, we monitored the kinetics of ASC and HRMVPC interaction with HG-conditioned EC ECM by measuring the percent of cell confluence (Supplementary Figure S2). The increase of ASC confluence was higher on EC ECM compared to culture plastic (Figure 2A). The HUVEC ECM, conditioned with NG, HG, or mannitol, did not significantly affect the ASC confluence. HG conditioning rather increased ASC confluence on HUVEC ECM, although non-significant (Figures 2C,D). Interestingly, the ASC-improved interaction was not observed when using HRMVEC-derived ECM, reflecting the microvasculature of the retina (Figure 2E). As expected, the interaction between HRMVPC and HG-conditioned EC ECM was reduced (Figures 2B–D), however, only significant when assessing HRMVEC ECM (*p* ≤ 0.05, Figure 2E). Nevertheless, compared to ASC, the primary HRMVPC showed reduced confluence levels, which were significant for the first 14 h when assessing the confluence on HUVEC ECM (*p* ≤ 0.05, Figure 2D) but not when assessing the confluence on HRMVEC ECM (Figure 2E).

The differing starting points of the kinetic analysis and the fact that the curves did not separate further over time suggested an early effect of the HG-conditioned ECM, most probably on cell adhesion. Unfortunately, up to now, we were not able to provide conclusive evidence for this. Measuring the cell spreading and counting the adherent cells 10 and 20 min after cell seeding indicated slight but non-significant differences between ASC and HRMVPC, but none when comparing NG- and HG-conditioned EC ECM. The adhesion kinetics, addressing focal adhesion dynamics as previously shown (Dreher et al., 2013), were also inconclusive. We also found no quantifiable measures of HG-induced ECM modifications: staining with an advanced glycation end product-specific antibody (kindly provided by Thomas Fleming, Heidelberg) was only detected in positive controls after methylglyoxal treatment. Quantifying sulfated glycosaminoglycans of NG- and HG-conditioned EC ECM revealed no differences either. Furthermore, no significant changes were found in gene expression upon comparing HRMVEC (not shown as only cells from one donor were tested) and HUVEC cultured for 5 days in NG and HG conditions (Supplementary Figure S4C).

### Human ASC Are Pro-angiogenic Whereas HRMVPC Do Not Support Angiogenic Network Formation

**FIGURE 3:**
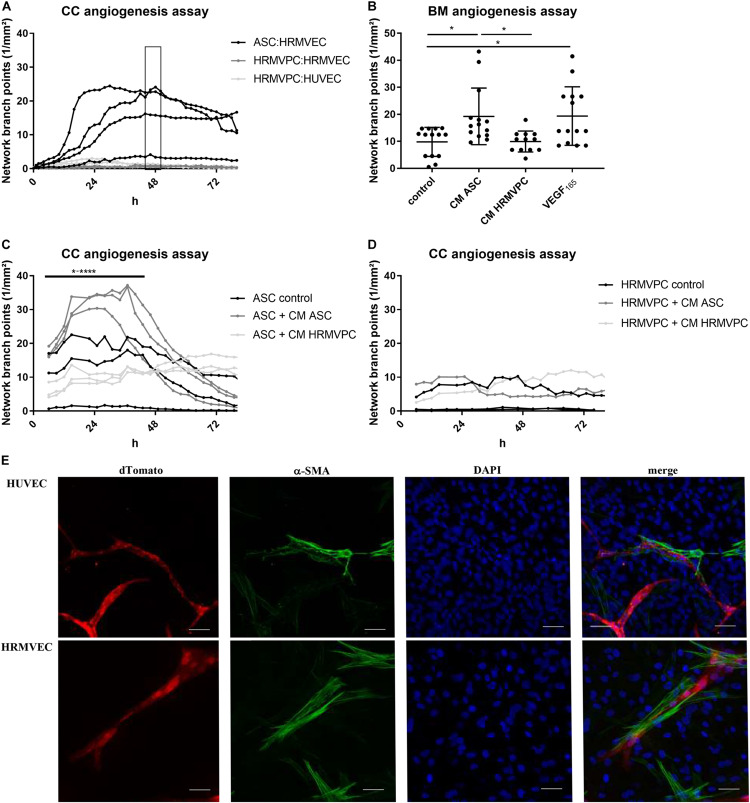
Pro-angiogenic activity discriminates human adipose-derived stromal cells (ASC) and retinal pericytes. **(A)** Vascular network formation in CC angiogenesis assay using network branch point metrics (1/mm^2^) of ASC/human retinal microvascular endothelial cells (HRMVEC), human retinal microvascular pericytes (HRMVPC)/HRMVEC, and HRMVPC/human umbilical vein endothelial cells (HUVEC) monitored using live imaging (four different ASC donors, two and three independent experiments with HRMVPC/HRMVEC and HRMVPC/HUVEC, respectively). Medium change is indicated by the box. ASC/HUVEC cocultures are not shown. **(B)** Vascular network formation in a BM angiogenesis assay (*n* = 11 paired experiments with ASC from 11 different donors and HRMVPC from three different donors in independent experiments; *p ≤ 0.05, one-way ANOVA with Tukey’s *post hoc* test). **(C,D)** Vascular network formation in CC angiogenesis assay, adding ¼ ECGM-2 as control or CC angiogenesis assay-derived CM ASC or CM HRMVPC to ASC/HRMVEC **(C)** or HRMVPC/HRMVEC **(D)** cocultures (*n* = 3, with three different ASC donors, independent experiments for two HRMVPC donors; *p ≤ 0.05 to ****p ≤ 0.0001, two-way RM-ANOVA with Tukey’s *post hoc* test). **(E)** Representative images showing the expression of α-smooth muscle actin (α-SMA) in ASC/EC cocultures (dTomato EC, red; α-SMA green; DAPI, blue nuclear counter stain and merge). Scale bar, 50 μm.

A key function of pericytes is to control endothelial cell angiogenesis or quiescence (Teichert et al., 2017). To compare how ASC and HRMVPC regulate angiogenesis, we established two kinetic angiogenesis assays *in vitro*—one focusing on cell–cell interactions (direct CC angiogenesis assay) and a second one addressing paracrine factors (BM angiogenesis assay). Both assays evaluate the endothelial cell network formation by live cell imaging (Supplementary Figure S3A). The ASC donor-dependently supported endothelial network formation by increasing the network branch points (Figure 3A) and the network length (Supplementary Figure S3B). This effect was transient, lasting for approximately 48 h, but could be maintained for 3 weeks upon regular medium exchange. In general, the levels of tube formation were stronger in HUVEC compared to HRMVEC (Fiori et al., 2020). In contrast to ASC, HRMVPC did not support network formation, neither of HRMVEC nor of HUVEC (Figure 3A). To determine whether this is related to cell–cell or paracrine factor, we compared the CM of ASC and HRMVPC (prepared from CC angiogenesis assays) to VEGF_165_ addition in the BM angiogenesis assay. CM ASC significantly induced network formation similar to VEGF_165_, whereas CM HRMVPC had no effect (*p* ≤ 0.05, Figure 3B). This indicates that ASC secrete angiogenic factors. Speculating that HRMVPC produce anti-angiogenic/angiostatic factors, we added CM from ASC or HRMVPC cocultures to CC angiogenesis assays, respectively. Indeed CM HRMVPC abolished the supportive function of ASC (*p* ≤ 0.05 to *p* ≤ 0.0001, Figure 3C). CM ASC was not capable of converting HRMVPC to support angiogenesis (non-significant, Figure 3D). Interestingly, the ASC from one donor that did not support vascular network formation in the control setting—the same donor as depicted in Figure 3A – supported tube formation once CM ASC was added. The ASC, which aligned to and wrapped around the endothelial tube-like structures, showed a strong expression of α-SMA, indicating a pericyte-like differentiation (Figure 3E).

These data clearly suggest that ASC exert a pro-angiogenic activity by providing a matrix and by secreting angiogenic factors, whereas HRMVPC do not support and—by secreting anti-angiogenic factors—actively inhibit endothelial tube formation.

### Gene and Protein Expression Profiling

**FIGURE 4:**
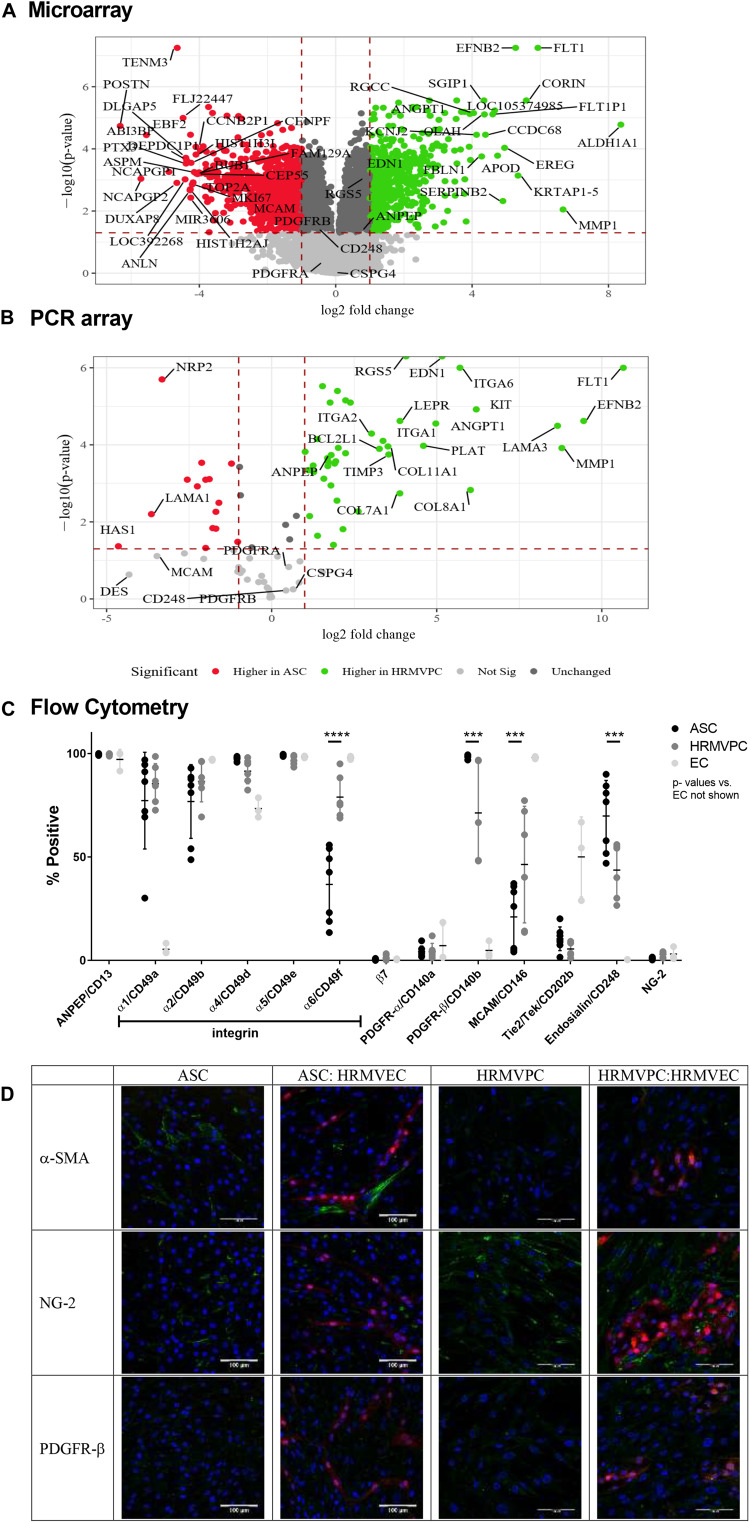
Differential marker expression of adipose-derived stromal cells (ASC) and human retinal microvascular pericytes (HRMVPC) assessed by microarray, PCR array, flow cytometry, and immunofluorescence. **(A)** Volcano plot visualizing the microarray data showing the magnitude of change (log_2_-fold change, *x*-axis) vs. statistical significance [-log_10_(*p*-value), *y*-axis] of gene expression of ASC vs. HRMVPC (each *n* = 3 biological replicates). Most differentially expressed genes and putative pericyte markers are labeled. **(B)** Validation of microarray results using PCR array. The correlation is high (Spearman correlation *R* = 0.95, *p* < 2.2e-16). Most differentially expressed genes and putative pericyte markers are labeled. **(C)** Marker expression in ASC, HRMVPC, and EC measured by flow cytometry. Percent positivity calculated against the unstained control [*n* = 7 different ASC donors, *n* = 6 HRMVPC with three donors in independent experiments, *n* = 3 human umbilical vein endothelial cells and human retinal microvascular endothelial cells (HRMVEC); ***p ≤ 0.001 and ****p ≤ 0.0001, two-way ANOVA with Tukey’s *post hoc* test, *p*-values for EC not shown]. Correlation to microarray and PCR array is poor (Spearman correlation *R* = 0.4, *p* = 0.2) using mean fluorescence intensity values. **(D)** Representative pictures of marker expression (green) in ASC, HRMVPC, and the respective cocultures measured by two-photon microscopy. dTomato HRMVEC are shown in red. Scale bar, 100 μm.

Our data suggested that the pro-angiogenic activity discriminates ASC from HRMVPC. To gain further insight into the potential contributing mechanisms, we performed whole-genome microarrays and validated data using PCR arrays, flow cytometry, and immunofluorescence analyses. The microarray gene expression profiling resulted in 2,533 genes more highly expressed in ASC and 2,873 higher in HRMVPC (Figure 4A). The KEGG pathway analysis revealed that, in ASC, the more highly expressed genes grouped to genetic information processing, whereas in HRMVPC, the more highly expressed genes grouped to environmental information processing. The selected candidate genes were validated using a PCR array (Figure 4B). There was a strong correlation between the microarray and the PCR array data (Spearman *R* = 0.95, *p* < 2.2e-16).

In order to understand the different adhesion of ASC and HRMVPC to the HG-conditioned EC ECM, we first zoomed into cell adhesion-related genes (based on a NCBI gene query with “cell adhesion AND ‘Homo sapiens’ [porgn:__txid9606]”) and found 434 differentially expressed genes. The integrin subunits alpha 1, 2, 4, and 6 (*ITGA 1*, *2*, *4*, and *6*) appeared to be the most upregulated genes in HRMVPC, whereas ASC expressed integrin subunits alpha 5 and beta 3 (*ITGA5* and *ITGAB3*) to a higher level (Supplementary Figures S4A,B). Accordingly, we assessed the surface expression of integrins by flow cytometry. However, only integrin alpha-6 (CD49f) was heterogeneously expressed, higher in HRMVPC (69–95%) than in ASC (13–56%) (*p* ≤ 0.0001, Figure 4C).

With respect to genes related to ECM, laminin subunit a3 (*LAMA3*), collagen type VIII alpha 1 chain (*COL8A1*), and collagen type XI alpha 1 chain (*COL11A1*) were more highly expressed in HRMVPC, whereas ASC showed a high expression of laminin subunit alpha 1 (*LAMA1*), tenascin C (*TNC*), and collagen type VI alpha 1 and 2 (*COL6A1* and *COL6A2*) (Supplementary Figures S4A’,B’). The HRMVPC expressed matrix metalloproteinase-1 (*MMP1*) and metalloproteinase inhibitor 3 (*TIMP3*), which are involved in EC tubular morphogenesis, at significantly higher levels than ASC did.

Finally, we studied the expression of common pericyte markers. Some of the HRMVPC-specific genes appeared with high standardized value scores, indicating relative strength with functional association, within the gene set “pericytes” of the database Harmonizome (Rouillard et al., 2016), such as VEGF receptor 1 (*FLT1*), endothelin (*EDN1*), *RGS5, MMP1*, angiopoietin-1 (*ANGPT1*, Ang-1), and fibulin-1 (*FBLN-1*). None of the ASC-specific genes scored here (Figure 4A). Comparing gene expression with protein expression data (flow cytometric MFI values) gave low correlation values (Spearman correlation *R* = 0.4, *p* = 0.2) in line with previous reports (Vogel and Marcotte, 2012). The alanyl aminopeptidase (*ANPEP/*CD13) mRNA levels, for instance, appeared to be increased in HRMVPC, whereas the flow cytometry data showed no significant differences in protein expression. In general, flow cytometry revealed a considerable heterogeneity in both ASC and HRMVPC cultures. The percentage of melanoma cell adhesion molecule (CD146)-positive cells was larger within HRMVPC than in ASC (46 ± 28% vs. 21 ± 15%, *p* ≤ 0.001, Figure 4C), whereas endosialin (CD248) was expressed by a larger fraction of ASC (70 ± 17% vs. 44 ± 13%, *p* ≤ 0.001, Figure 4C). PDGFR-β (CD140b), generally considered to be a pericyte marker, was expressed by 98 ± 1% of ASC, whereas in HRMVPC cultures the values varied (71 ± 24%, *p* ≤ 0.001, Figure 4C). PDGFR-β expression was weak in both ASC and HRMVEC after immunofluorescence staining and independent of angiogenic EC coculture (Figure 4D). In addition, the common pericyte marker NG-2 was assessed (Bergers and Song, 2005) to validate the array data in which NG-2 did not appear as a differentially expressed gene. NG-2 was positive with immunofluorescence, more in HRMVPC than in ASC, independent of EC coculture (Figure 4D), but not detectable by flow cytometry [not detectable by extra- or intracellular staining, most probably related to trypsinization (Schmitt et al., 2018)]. As shown before, those ASC wrapping around the tubular structures in the CC angiogenesis assay were strongly positive for α-SMA (Figure 4D). In line with the lack of angiogenenic tube formation, this did not occur in HRMVPC/HRMVEC CC angiogenesis cultures. In monocultures, α-SMA expression was weak and restricted to a fraction of ASC and also of HRMVPC. The gene expression data clearly demonstrated RGS5 as a differentially expressed pericyte marker (Figure 4A). However, we were not able to assess RGS5 protein expression by flow cytometry or immunofluorescence since negative results were obtained even in the expected positive controls.

Overall these data demonstrate marked differences in gene expression profiles and document a considerable degree of heterogeneity in ASC and HRMVPC cultures, with an overlap of the expressed markers.

### Conditioned Medium Composition Differs Significantly Between ASC and HRMVPC

To explain the different effects of CM ASC and HRMVPC within the angiogenesis assays, we further addressed the differential gene expression of secreted angiogenic and anti-angiogenic/angiostatic factors. Zooming into angiogenesis-related genes (based on a NCBI gene query with “angiogenesis AND ‘Homo sapiens’ [porgn:__txid9606]”), we found 450 differentially expressed genes within the microarray data sets. *ANGPT1*, *EDN1*, ephrin-B2 (*EFNB2*), Bcl-2-like 1 (*BCL2L1*), and transforming growth factor-beta 2 (*TGFB2*) stood out in HRMVPC, whereas interleukin 6 (*IL-6*) and *VEGF-A* were most upregulated in ASC (Supplementary Figures S4A”,B”). *TGFB1* and *TGFB3* were also higher in ASC, but at the significant limit.

**FIGURE 5:**
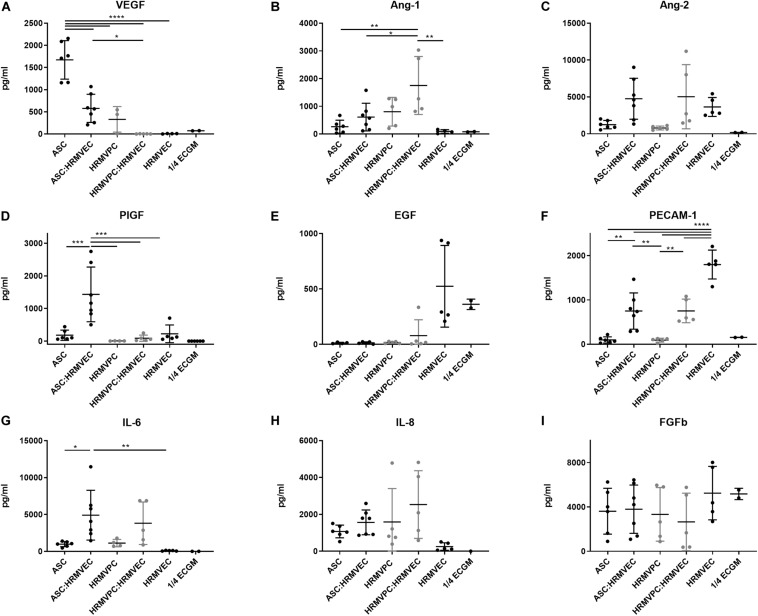
Conditioned medium composition differs significantly between adipose-derived stromal cells (ASC) and human retinal microvascular pericytes (HRMVPC). Angiogenic growth factor levels **(A–I)** of conditioned cell culture supernatants (CM) of mono- or cocultures measured by flow cytometry-based plex assay (*p ≤ 0.05, **p ≤ 0.01, ***p ≤ 0.001, and ****p ≤ 0.0001, one-way ANOVA with Tukey’s *post hoc* test) (ASC from six different donors, HRMVPC from two different donors in independent experiments, ¼ ECGM-2 *n* = 2 independent experiments).

To validate these results, we quantified the angiogenesis-related growth factors in the CM of ASC and HRMVPC mono- and CC angiogenesis cocultures. We observed some donor-dependent variability, but a paired analysis revealed significant changes that support the gene expression data. In fact, ASC showed a significantly higher production of VEGF in monocultures, which decreased in cocultures, indicating its use (*p* ≤ 0.0001, Figure 5A). The values exceeded those in HRMVPC cultures (*p* ≤ 0.05). The Ang-1 levels, in contrast, were highest in HRMVPC cocultures, which were 2.8-fold higher than in the ASC cocultures (*p* ≤ 0.05, Figure 5B). The Ang-2 levels increased in both ASC and HRMVPC cocultures compared to those in the monocultures (3.8- and 6.3-fold, respectively, non-significant, Figure 5C). Placental growth factor (PlGF), low in all other conditions, was significantly higher in the ASC cocultures (*p* ≤ 0.001, Figure 5D). The levels of epidermal growth factor (EGF) were high in HRMVEC monocultures but low in all other conditions (non-significant, Figure 5E). The PECAM-1 levels were highest in HRMVEC monocultures, significantly reduced in cocultures, and very low in ASC and HRMVPC monocultures (*p* ≤ 0.01, Figure 5F). The IL-6 levels were significantly increased in ASC cocultures compared to their respective monocultures (*p* ≤ 0.05, Figure 5G). IL-8 and fibroblast growth factor-beta (FGFb) remained unaffected by the culture condition (Figures 5H,I). Importantly, only EGF and FGFb were detectable in ¼ ECGM-2 tested as control. These data suggest that VEGF and PlGF are potential candidates for the observed ASC pro-angiogenic potential, and Ang-1 is a candidate for the HRMVPC anti-angiogenic/angiostatic effects.

### VEGFR-2 Inhibition Efficiently Abolishes ASC Angiogenic Supportive Capacities

**FIGURE 6:**
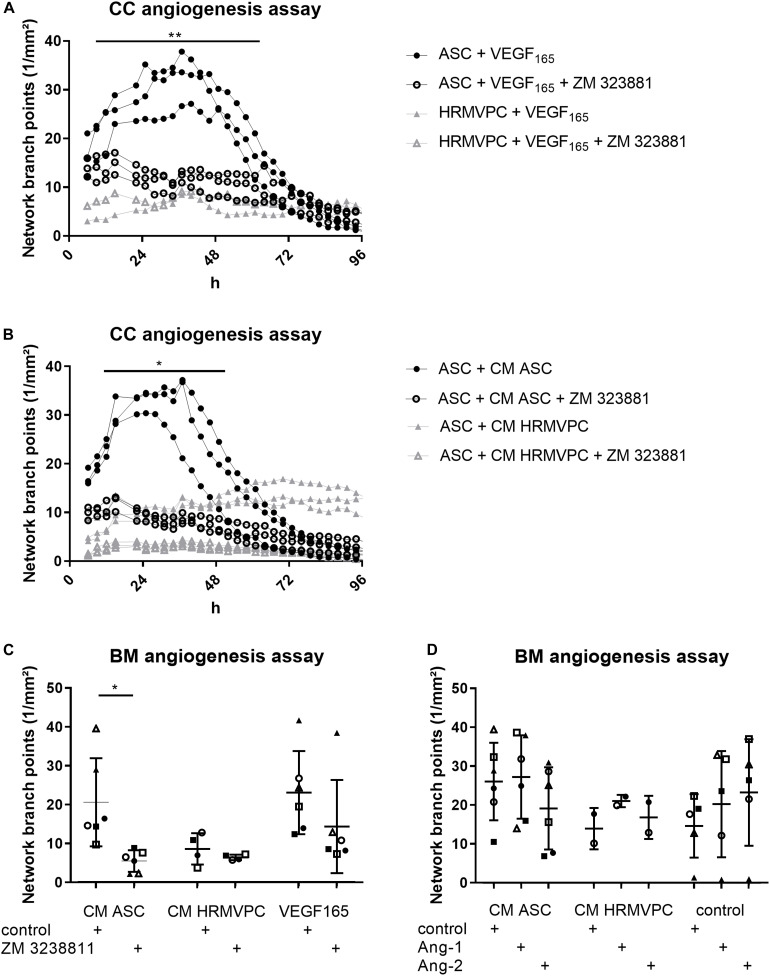
VEGFR-2 inhibition efficiently abolishes adipose-derived stromal cells (ASC) angiogenic supportive capacities, whereas Ang-1 and Ang-2 addition do not alter effects. **(A)** Vascular network formation in CC angiogenesis assay. VEGF and the VEGF inhibitor ZM 3238811 were added to ASC-human retinal microvascular endothelial cell (HRMVEC) or human retinal microvascular pericyte (HRMVPC)/HRMVEC cocultures (three different ASC donors and one HRMVPC donor; **p ≤ 0.01, two-way RM-ANOVA with Tukey’s *post hoc* test). **(B)** Vascular network formation in CC angiogenesis assay. CM ASC or CM HRMVPC are added to ASC/HRMVEC cocultures with the VEGF inhibitor ZM 323881 (three different ASC donors; *p ≤ 0.05, two-way RM-ANOVA with Tukey’s *post hoc* test). **(C)** Vascular network formation in BM angiogenesis assay assessing CM ASC, CM HRMVPC, and VEGF_165_ in the absence and the presence of the VEGF inhibitor ZM 323881 (different ASC donors indicated by different symbols; *p ≤ 0.05, two-way ANOVA with Sidak’s *post hoc* test). **(D)** Vascular network formation in BM angiogenesis assay assessing CM ASC, CM HRMVPC, and control condition in the absence and the presence of recombinant Ang-1 or Ang-2 (different ASC donors or independent HRMVPC experiments indicated by different symbols; non-significant, two-way ANOVA with Tukey’s *post hoc* test).

Especially the high levels of VEGF in ASC/HRMVEC cocultures and Ang-1 in HRMVPC cocultures are in line with their known pro- and anti-angiogenic effects and prompted us to investigate these in more detail. As observed previously (Figure 3B), VEGF_165_ had similar effects as CM ASC in promoting tube formation (Figures 6A,C). Using suramin as an inhibitor of angiogenesis, the BM matrix dissolved and EC grew to monolayers without forming tube-like structures (not shown). Similar observations were made previously (Prigozhina et al., 2013). Therefore, instead of using suramin, we added the selective VEGFR-2 inhibitor ZM 3238811, which abolished the supportive effect of VEGF_165_ in the ASC coculture angiogenesis assay (*p* ≤ 0.05, Figure 6A). ZM 3238811 likewise significantly abolished the supportive effect of CM ASC (*p* ≤ 0.05, Figures 6B,C). As expected, HRMVPC showed no effect on EC tube formation and thus were rather unaffected by the VEGFR-2 inhibitor. Interestingly, in the CC angiogenesis assays on ASC, the inhibitor reduced the network formation affected by CM HRMVPC even further. The inhibitory effect of CM HRMVPC again appeared to be only transient because the branch point values started to increase slightly after 48 h in the ASC cocultures (Figure 6B). We verified that the inhibitor did not compromise EC viability. These data suggest that VEGF/VEGFR-2 signaling is required for the pro-angiogenic function of ASC.

We considered Ang-1 as an anti-angiogenic effector in CM HRMVPC. However, both Ang-1 and Ang-2—used as control—slightly increased tube formation in the control medium (non-significant, Figure 6D). Ang-1 failed to reduce the pro-angiogenic function of CM ASC. In only one culture (Δ), Ang-1 inhibited the pro-angiogenic effect of CM ASC. Other CM ASC showed an even increased angiogenic effect upon adding Ang-1. A similar trend was also found in HRMVPC, where Ang-1 led to a small increase in branching tubular networks. These data suggest that HRMVPC use other/further anti-angiogenic/angiostatic factors than just Ang-1.

## Discussion

Diabetic retinopathy is a frequent diabetes-associated complication. Endothelial dysfunction ensues due to pericyte loss. ASC have been suggested as protective and regenerative cellular therapy and functional replacement of pericytes. Indeed the use of ASC in several different animal models of DR brought positive results, preventing retinal degeneration and vessel dysfunction. However, the exact mode of action of MSC in the retinal microenvironment is still unclear. The pericyte-like function and the pro-angiogenic potential are the most likely candidates. To support evidence for this, we compared the pro-angiogenic and the pericyte-like features of ASC and HRMVPC. Our results showed that ASC and HRMVPC: (1) differ in morphology, growth potential, and gene and protein expression (Figures 1, 4), (2) show marked heterogeneity with significantly different percentages of marker-positive cells (Figure 4), (3) exhibit different interaction with HG-conditioned EC ECM, mimicking the DR microenvironment (Figure 2), and (4) can be clearly discriminated by their angiogenic activity, which involves VEGF, but probably not Ang-1 (Figures 3–6). These properties support the use of ASC as cell-based therapy in DR but also raise potential concerns that call for a careful risk-to-benefit analysis.

### Distinction Between ASC and HRMVPC

Until now, there is no clear distinction possible between MSC/ASC and pericytes. There is an intense discussion about the *in vivo* identity of MSC that appear to reside in a perivascular location similar to pericytes (Meirelles et al., 2006; Crisan et al., 2008). Perivascular cells express both MSC markers and pericyte markers. Functional tests led to regard MSC as progenitors of pericytes (Crisan et al., 2008; Meirelles et al., 2015; da Silva Meirelles et al., 2016; de Souza et al., 2016; Guimaraes-Camboa et al., 2017; Hardy et al., 2017). Comparing ASC and HRMVPC, we observed differences in morphology and growth potential. In addition, we found a set of differentially expressed genes with a good correlation between microarray and PCR array results. The protein expression analyses, however, resulted in a poor correlation to gene expression, similar to previous reports (Vogel and Marcotte, 2012). Marker expression confirmed a distinct heterogeneity of cell cultures. Many markers were shared, but CD105, CD106, HLA-DR, CD49f, PDGFR-β, CD146, and CD248 revealed a significant variation in the percentage of marker-positive cells, which is in line with previous findings (Blocki et al., 2013; Vezzani et al., 2016).

We now provide evidence that the pro-angiogenic capacity discriminates ASC from HRMVPC. Hypothesizing that ASC may replace pericytes and restore microvascular support in DR, we first compared the interaction between ASC, HRMVPC, and EC ECM by recapitulating the DR-like environment. The significant reduced interaction of HRMVPC with HG-modified EC ECM has been taken as one possible explanation for the pericyte dropout seen in DR (Beltramo et al., 2009). We observed only a slightly impaired adhesion of HRMVPC and no signs of apoptosis. The ASC compared to HRMVPC, however, showed a significantly improved interaction with HG-modified EC ECM. As shown in a previous study (Fiori et al., 2020), the growth factors in the ECGM-2 medium protected EC from HG effects, which is why we reduced their concentration to one quarter. However, despite lowering the concentration, gene expression showed no significant changes between the NG and the HG-cultured EC. Various further analyses did not help to identify the molecular basis for HG-induced ECM changes and the differing interaction of ASC and HRMVPC. Only by using multiple particle tracking microrheology were we able to observe HG-mediated changes in the elasticity of HUVEC ECM, suggesting that the HG effect might relate to the altered mechanical properties of the ECM (Hafner et al, 2020). Whether this is related to physical processes, like osmotic shrinking, or chemical modifications, like glycation, remains to be investigated.

Comparing next the angiogenic properties of ASC and HRMVPC, we showed that the vascular network formation assays can clearly discriminate ASC from HRMVPC. Whereas the ASC induced tube formation, the HRMVPC lacked this support and even actively inhibited network formation, both in a paracrine fashion. The gene expression data supported our observations, indicating a pro-angiogenic profile for ASC and an anti-angiogenic one for HRMVPC.

In CC angiogenesis assays, the ASC underwent a pericyte-like differentiation, characterized by α-SMA-positive cells aligning to the tube-like structures, as shown previously (Merfeld-Clauss et al., 2014). These authors identified that, besides cell–cell contacts, ASC-derived VEGF and HGF and EC-derived PDGF-BB were essential for vascular network formation and linked activin-A to the localized α-SMA expression (Merfeld-Clauss et al., 2010, 2014).

The observed lack of HRMVPC to support tube formation is in line with the vessel-stabilizing function controlling EC sprouting, proliferation, and patterning of remodeling vascular networks (Orlidge and Damore, 1987; Simonavicius et al., 2012). Supporting our findings, Bodnar et al. (2013) described that pericytes, isolated from skeletal muscles, actively prevented basal lamina matrix-induced vessel formation and even induced regression of preformed vascular structures. Contrasting data, however, describe the co-assembly of EC and pericytes in 3D collagen matrices (Stratman et al., 2009). In this serum-free model, hematopoietic growth factors, such as stem cell factor, IL-3, and stromal-derived factor a, were added, which may explain the observed differences. Furthermore, Blocki et al. (2013) reported that placental pericytes were best in supporting and stabilizing the morphologically intact tube structures, comparing pericytes from different tissues, MSC and CD146(+)CD34(-) and CD146(-) BM-MSC. This may suggest that either culture conditions or source and phenotype/marker expressions define function. Interesting candidates for this may be the differentially expressed markers identified in our study. CD248/endosialin, more strongly expressed in HRMVPC than in ASC, has been linked to pericyte-mediated vascular patterning (Simonavicius et al., 2012). The α6β1 integrin/CD49f appears to be required for perivascular localization and α-SMA expression (Carrion et al., 2010, 2013). CD146 expression on MSC seems to be essential for basal lamina matrix-induced tube formation as only CD146(+)CD34(-) MSC stabilized endothelial networks and improved endothelial sprout integrity (Blocki et al., 2013).

Our data hint that soluble factors contribute to the pro- and anti-angiogenic activities of ASC and HRMVPC. We observed that the paracrine anti-angiogenic effect of CM HRMVPC appeared to be transient. The performed growth factor analysis suggested VEGF and PlGF as potential pro-angiogenic factors, significantly enriched in CM from ASC mono- and cocultures, and Ang-1 as potential anti-angiogenic factor in CM from HRMVPC cocultures. Indeed the inhibition of VEGFR-2 abolished the supportive effect of ASC and CM ASC on tube formation, indicating that VEGF/VEGFR-2 signaling is required. Furthermore, in the ASC/HRMVEC coculture, VEGFR-2 inhibition added to the CM HRMVPC effect, confirming the importance of VEGF. In line with this, VEGFR-1 (Flt1) may be involved in mediating the CM HRMVPC anti-angiogenic effect. Flt1, particularly its soluble form, is known to antagonize VEGF–VEGFR2 interaction, and by this, to modulate EC behavior (Eilken et al., 2017), *FLT1* mRNA was significantly more highly expressed by HRMVPC than by ASC.

However, we considered it more likely that Ang-1 is involved because Ang-1–Tie2 signaling is known as an important regulator of the maintenance of a quiescent EC phenotype (Teichert et al., 2017). The significantly higher levels of Ang-1 in HRMVPC cocultures suggested an anti-angiogenic function. However, tube formation was not altered upon adding rhAng-1 or rhAng-2 as control, neither in the control nor in the CM ASC setting. Given the known high complexity and plasticity of pericyte-mediated control of angiogenesis (Teichert et al., 2017), we decided to focus on CM ASC.

We observed a clear ASC donor-dependent level in tube formation, in line with our previous study. In this, we correlated the growth factor concentrations in CM of ASC to the level of vascular network support (Fiori et al., 2020). The levels of all growth factors, except for Ang-1 and VEGF, correlated positively to tube formation. Ang-1 correlated negatively and VEGF showed no correlation, which was surprising given the data shown herein that VEGF appears as the most relevant factor for ASC’s pro-angiogenic activity. Unfortunately, in this study, it was not possible to perform similar correlation calculations and especially to analyze the one ASC donor that repeatedly lacked pro-angiogenic activity on its own. Taking both studies together, we propose that ASC secrete VEGF in excess and that the concentration of FGFb, PECAM-1, PlGF, Ang-1, and Ang-2—probably set off against each other—makes up the level of tube formation. In line with this, Lehman et al. (2012) defined the concentrations of 35 pg/ml VEGF, 110 pg/ml IL-8, and 2,050 pg/ml CXCL-5 to be required for angiogenesis induction. In our setting, the levels of both VEGF and IL-8 were higher in ASC mono- and cocultures than these reported minimal concentrations. Although not yet addressed, PlGF appears to be a highly interesting candidate for further investigations. PlGF was found to be increased in ASC derived from patients with coronary artery disease and diabetes mellitus type 2. These, however, exerted significantly reduced angiogenic activity due to increased anti-angiogenic factors (Dzhoyashvili et al., 2014). These data suggest a disturbed homeostasis of pro- and anti-angiogenic factors in these patients.

### ASC for Cell-Based Therapy of DR

The underlying question for this study was whether ASC can functionally replace HRMVPC and restore vascular stabilization in DR. As previously introduced, ASC treatment improved visual function and delayed DR progression (Yang et al., 2010; Mendel et al., 2013; Rajashekhar et al., 2014; Ezquer et al., 2016). *In vitro* data from this and our previous studies support this since ASC (1) resist HG-stress (Hajmousa et al., 2016; Fiori et al., 2020), (2) reverse high glucose-induced reduction of angiogenesis in HRMVEC, probably by reducing the oxidative stress levels (Fiori et al., 2020) or the HG-induced proinflammatory activation of EC (Hajmousa et al., 2018), (3) show improved interaction with HG-conditioned HUVEC ECM compared to HRMVPC (shown here), (4) have a pro-angiogenic activity which discriminates them from HRMVPC (shown here), and finally (5) act as functional pericyte-like cells *in vivo* (Hajmousa et al., 2018; Terlizzi et al., 2018).

However, the pro-angiogenic activity of ASC may pose a certain risk for ASC-based therapies. The angiogenic factors are crucial for DR development and progression, with VEGF being the most relevant. That is why ocular anti-VEGF therapy has been introduced (Stitt et al., 2016). In 2017, three patients have been reported to become blind after adipose “stem cell” injection for age-related macular disease treatment (Kuriyan et al., 2017). Ocular hypertension, hemorrhagic retinopathy, vitreous hemorrhage, combined traction, and rhegmatogenous retinal detachment have been reported. Furthermore, in a non-diabetic transgenic model with damage of the neurovascular unit in the retina, intravitreal MSC injection, in fact, worsened the vascular damage by inducing cataract, increasing the loss of pericytes with subsequent retinal vasoregression, and provoking inflammatory responses (Huang et al., 2019). Furthermore, MSC-derived extracellular vesicles have been shown to impair the pericyte-stabilizing function by inducing pericyte detachment, migration, and angiogenesis *in vitro* (Beltramo et al., 2014). Non-clinical data, however, argue in favor of ASC safety and efficacy, showing that MSC readily adapt to the local milieu and orchestrate repair depending on the needs by secreting either pro- or anti-angiogenic factors. Ezquer et al. (2016) at least observed no signs of increased intraocular angiogenic growth factors, rather of the anti-angiogenic factor thrombospondin 1, and no effect on the blood vessels after ASC injection. In a model of corneal wound healing, locally administered MSC likewise efficiently reduced the angiogenic and the inflammatory processes (Oh et al., 2008). Most importantly, Mendel et al. (2013) observed that the ASC therapeutic effects were exactly the needed ones: after vessel destabilization, ASC promoted vessel regrowth, whereas pretreatment with ASC prevented capillary dysfunction. As our observations are based only on *in vitro* experiments, we consider it imperative to investigate the therapeutic mode of action and especially the timing of cell application in suitable non-clinical models in more detail to assure a safe and efficacious therapy. It is also critical to address the question whether to use autologous or allogeneic cells as Cronk et al. (2015) already documented that ASC from diabetic animals have impaired function compared to their healthy counterparts. Furthermore, it would be interesting to understand whether the pro-angiogenic activity is shared by the whole ASC population or belongs to certain subpopulations as already suggested (Blocki et al., 2013; Sherman et al., 2017; Wang et al., 2018). Defining marker combinations that attribute to the observed function may help to fine-tune the therapeutic activity of ASC. The subtypes with increased angiogenic function could be better suited for wound healing approaches, whereas the subtypes with stabilizing features might be advantageous for DR or tumor treatments. Our assay platform, in line with the gene and the protein expression data sets, offers a valuable basis for expansion.

In summary, our data support the use of ASC as candidates for a cell-based therapy in early stages of DR, suggesting that they can replace pericytes and even counteract vasoregression through the secretion of pro-angiogenic factors, mainly VEGF. However, the ASC-mediated pro-angiogenic activity may pose a risk to disease progression toward proliferative DR. Thus, a deeper study of the pro-angiogenic capacity of ASC may represent the turning point in the development of cell-based approaches in the treatment of DR. We suggest a careful risk-to-benefit analysis, including the characterization of the pro-angiogenic factors and their interaction and the regulation of their secretion, together with the definition of the optimal time point for ASC application, to take full advantage of the ASC therapeutic features.

## Data Availability Statement

The datasets generated for this study can be found in the GSE144605.

## Ethics Statement

The studies involving human participants were reviewed and approved by Mannheim Ethics Commission II. The patients/participants provided their written informed consent to participate in this study.

## Author Contributions

HeK, JG, KB, and AF contributed to the conception, design, acquisition, analysis, interpretation of data, and drafting of the manuscript. SU, EB, LS, MH, and CS contributed to the design and material, performed the analysis, and interpretation of the certain data. H-PH, MH, and HaK contributed to the conception, design, and financial contribution. All authors contributed to the manuscript revision and read and approved the submitted version.

## Conflict of Interest

The authors declare that the research was conducted in the absence of any commercial or financial relationships that could be construed as a potential conflict of interest.

## Footnotes

1 http://www.genome.jp/kegg

2 http://www.ncbi.nlm.nih.gov/geo/

## Supplementary Material

FIGURE S1 Workflow for kinetic analysis of cell proliferation using IncuCyteZOOM. 1. Using the IncuCyteZOOM live imaging device, phase contrast images of cells are automatically collected at defined time intervals (contrast and brightness of micrographs adapted for better visibility). In general, 4 to 8 replicates are run. 2. With an automated tool, a segmentation mask is assigned that closely resembles the actual cell size, morphology and confluence state. This mask/processing definition can be refined and optimized on different selected micrographs. 3. Using this processing definition, the confluence analysis mask is cross-checked on individual micrographs for each time point and of different experiments and if required further optimized. 4. Kinetic plots of respective analyses metrics are finally generated by the IncuCyte software and results depicted as phase object confluence in percent for each time point. Raw data are exported for further evaluation. Visual confirmation is possible via export of images and movies.

FIGURE S2 Workflow for analyzing the interaction of ASC and HRMVPC with HG-conditioned EC ECM. 1. Using the IncuCyteZOOM, the proliferation of seeded EC, cultured under NG or HG conditions, is followed (contrast and brightness of micrographs increased). Upon reaching 100% confluence, the ECM is prepared by lysing the EC. 2. Intactness of the ECM is verified by brilliant blue stain. 3. Then ASC or HRMVPC are seeded on the ECM (contrast and brightness of micrograph increased). 8 replicates were run.4. By using an optimized segmentation mask/processing definition, 5. the adhesion and proliferation kinetics are analyzed for each time point using different metrics: number of cells/image, average size of cells and percent confluence, respectively. 6. To allow for quantitative comparison between ASC and HRMVPC, confluence values were normalized against the respective NG-modified EC ECM control, values set as 1.

FIGURE S3 Kinetic analyses of angiogenesis assays. **(A)** 1. To detect network formation, phase contrast and fluorescence images are automatically recorded at defined time intervals in the IncuCyteZOOM using the tiled field of view (FOV) imaging mode. 3 to 8 replicates were run. 2. Using the integrated Angiogenesis Analysis Module, the fluorescence signal is used to quantify assay metrics: tube length and branch points for each time point. 3. The angiogenesis algorithm assigns a segmentation mask to resemble the vascular network. Exemplary micrographs depict network formation in CC angiogenesis (3A-C) and BM angiogenesis assay (4A, B). 5. Finally, kinetic data of angiogenesis metrics are plotted and exported for further evaluation (5A, B). **(B)** Comparison of network branch points and network length used as metrics to quantify network formation.

FIGURE S4 Differential gene expression of ASC and HRMVPC, and HUVEC cultured under normal or high glucose conditions. **(A)** Volcano plots visualizing microarray data depicting statistical significance (-log10(p-value), y-axis) versus magnitude of change (log2fold change, x-axis) of gene expression of ASC versus HRMVPC zooming into categories adhesion **(A)**, ECM **(A’)** and secreted factors **(A”)**, each n = 3 biological replicates. **(B)** Corresponding volcano plots of PCR array data used for validation of microarray data, separating the same categories: adhesion **(B)**, ECM **(B’)** and secreted factors **(B”)**, each n = 3 biological replicates. There was an overall high correlation between microarray and PCR array data (Spearman correlation R = 0.95, p ¡ 2.2e−16). **(C)** Volcano plot of PCR array data comparing HUVEC cultured for 5d in normal (NG) and high glucose (HG) conditions, n = 3 biological replicates, non-significant. Volcano plots were generated using the R package ggplot2. Similar data were obtained with HRMVECs (not shown, as only n = 1 biological replicate was analyzed in 3 independent experiments).

TABLE S1 Antibodies used for flow cytometry AF- Alexa Fluor, APC- Allophycocyanin, FITC- Fluorescein isothiocyanate, PE- Phycoerythrin.

TABLE S2 Gene list, Custom RT2 PCR Array.
