# Supplementary figures and images for: Pro-angiogenic Activity Discriminates Human Adipose-Derived Stromal Cells From Retinal Pericytes: Considerations for Cell-Based Therapy of Diabetic Retinopathy
Source: Front Cell Dev Biol. 2020 Jun 9;8:387. doi: 10.3389/fcell.2020.00387 (PMC7295949; doi:10.3389/fcell.2020.00387)

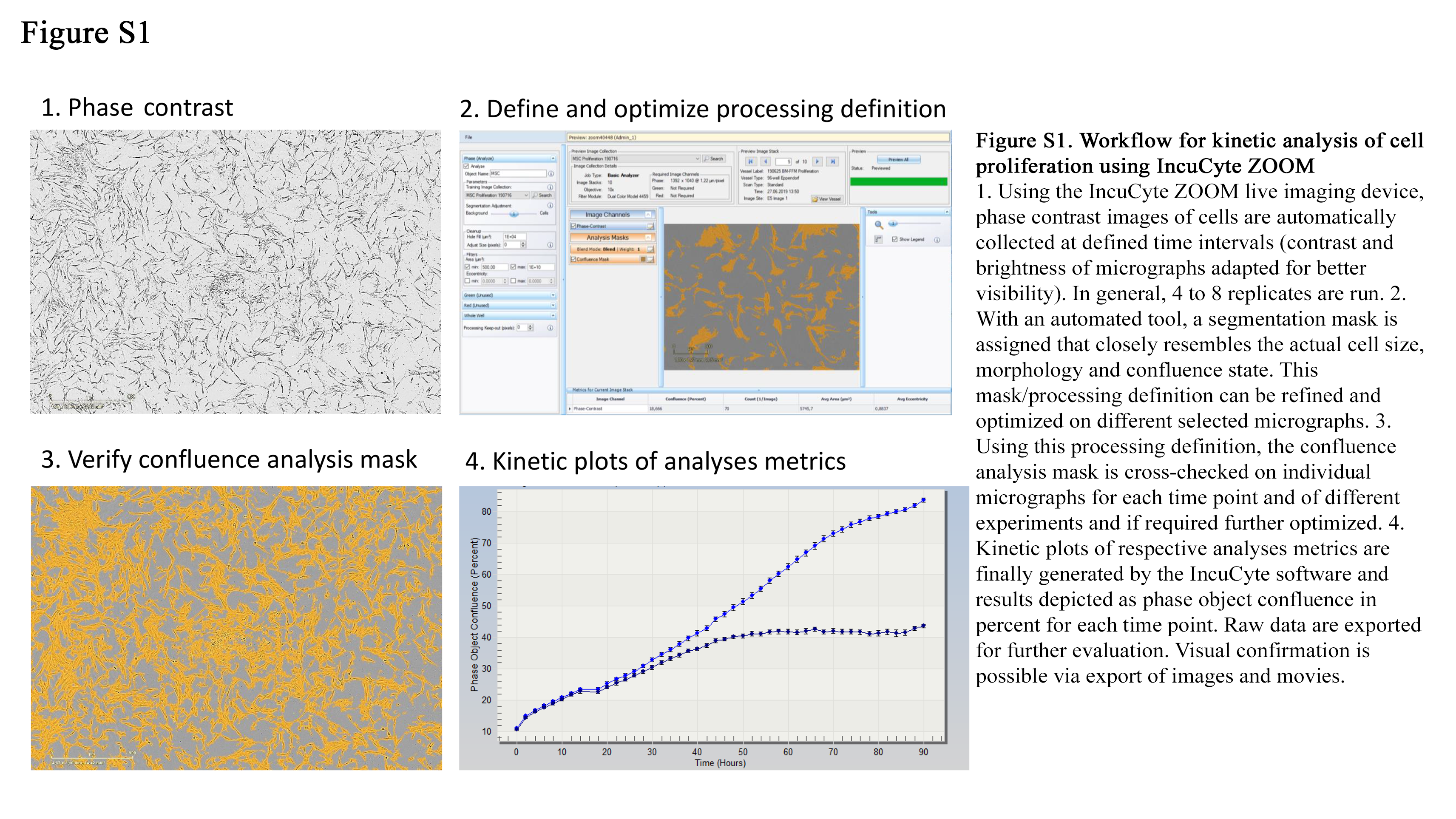

Supplement: FIGURE S1 — Workflow for kinetic analysis of cell proliferation using IncuCyteZOOM. 1. Using the IncuCyteZOOM live imaging device, phase contrast images of cells are automatically collected at defined time intervals (contrast and brightness of micrographs adapted for better visibility). In general, 4 to 8 replicates are run. 2. With an automated tool, a segmentation mask is assigned that closely resembles the actual cell size, morphology and confluence state. This mask/processing definition can be refined and optimized on different selected micrographs. 3. Using this processing definition, the confluence analysis mask is cross-checked on individual micrographs for each time point and of different experiments and if required further optimized. 4. Kinetic plots of respective analyses metrics are finally generated by the IncuCyte software and results depicted as phase object confluence in percent for each time point. Raw data are exported for further evaluation. Visual confirmation is possible via export of images and movies. [file Image_1.TIF]

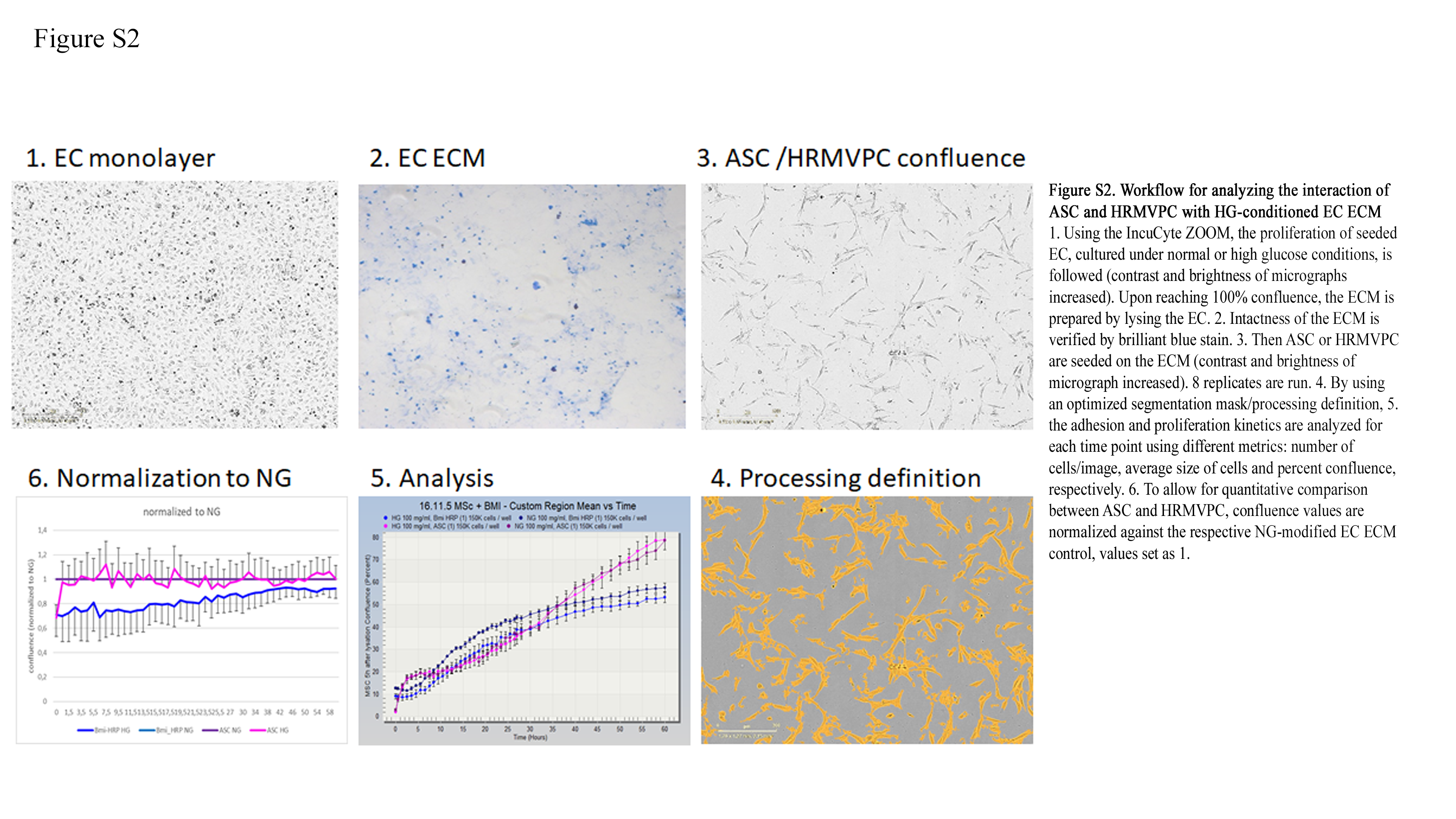

Supplement: FIGURE S2 — Workflow for analyzing the interaction of ASC and HRMVPC with HG-conditioned EC ECM. 1. Using the IncuCyteZOOM, the proliferation of seeded EC, cultured under NG or HG conditions, is followed (contrast and brightness of micrographs increased). Upon reaching 100% confluence, the ECM is prepared by lysing the EC. 2. Intactness of the ECM is verified by brilliant blue stain. 3. Then ASC or HRMVPC are seeded on the ECM (contrast and brightness of micrograph increased). 8 replicates were run.4. By using an optimized segmentation mask/processing definition, 5. the adhesion and proliferation kinetics are analyzed for each time point using different metrics: number of cells/image, average size of cells and percent confluence, respectively. 6. To allow for quantitative comparison between ASC and HRMVPC, confluence values were normalized against the respective NG-modified EC ECM control, values set as 1. [file Image_2.tif]

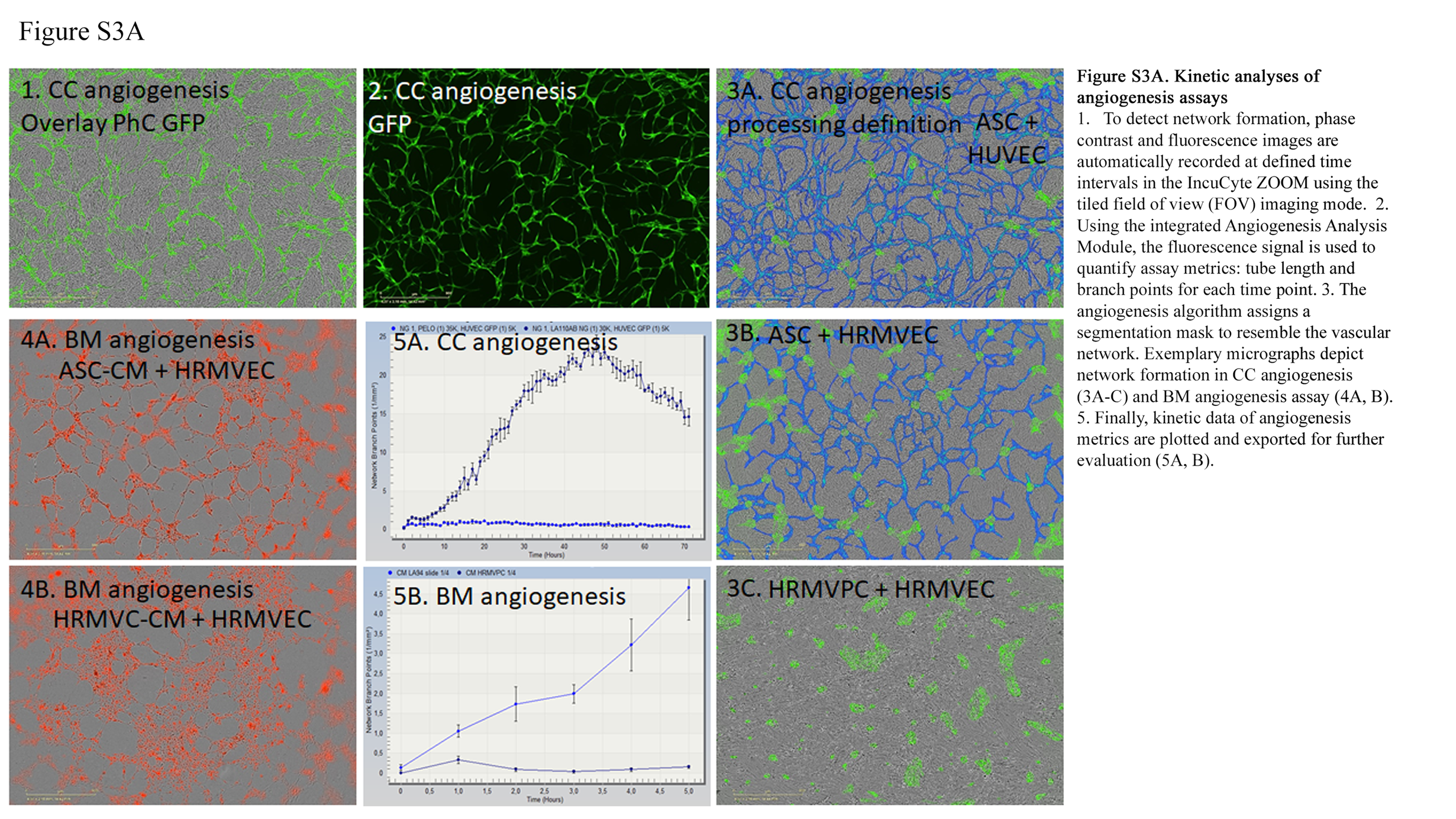

Supplement: FIGURE S3 — Kinetic analyses of angiogenesis assays. (A) 1. To detect network formation, phase contrast and fluorescence images are automatically recorded at defined time intervals in the IncuCyteZOOM using the tiled field of view (FOV) imaging mode. 3 to 8 replicates were run. 2. Using the integrated Angiogenesis Analysis Module, the fluorescence signal is used to quantify assay metrics: tube length and branch points for each time point. 3. The angiogenesis algorithm assigns a segmentation mask to resemble the vascular network. Exemplary micrographs depict network formation in CC angiogenesis (3A-C) and BM angiogenesis assay (4A, B). 5. Finally, kinetic data of angiogenesis metrics are plotted and exported for further evaluation (5A, B). (B) Comparison of network branch points and network length used as metrics to quantify network formation. [file Image_3.tif]

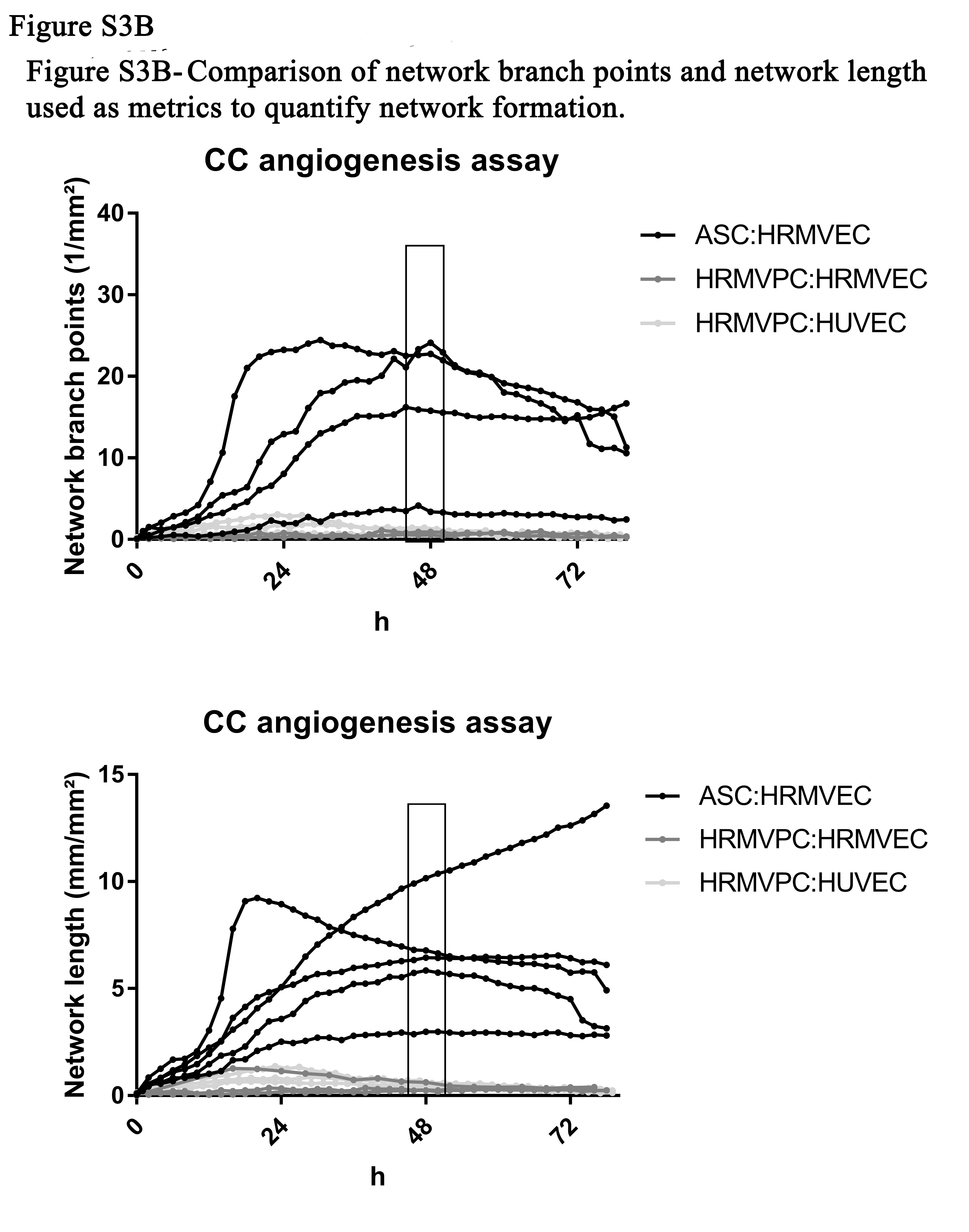

Supplement: FIGURE S4 — Differential gene expression of ASC and HRMVPC, and HUVEC cultured under normal or high glucose conditions. (A) Volcano plots visualizing microarray data depicting statistical significance (-log10(p-value), y-axis) versus magnitude of change (log2fold change, x-axis) of gene expression of ASC versus HRMVPC zooming into categories adhesion (A), ECM (A’) and secreted factors (A”), each n = 3 biological replicates. (B) Corresponding volcano plots of PCR array data used for validation of microarray data, separating the same categories: adhesion (B), ECM (B’) and secreted factors (B”), each n = 3 biological replicates. There was an overall high correlation between microarray and PCR array data (Spearman correlation R = 0.95, p ¡ 2.2e−16). (C) Volcano plot of PCR array data comparing HUVEC cultured for 5d in normal (NG) and high glucose (HG) conditions, n = 3 biological replicates, non-significant. Volcano plots were generated using the R package ggplot2. Similar data were obtained with HRMVECs (not shown, as only n = 1 biological replicate was analyzed in 3 independent experiments). [file Image_4.TIF]

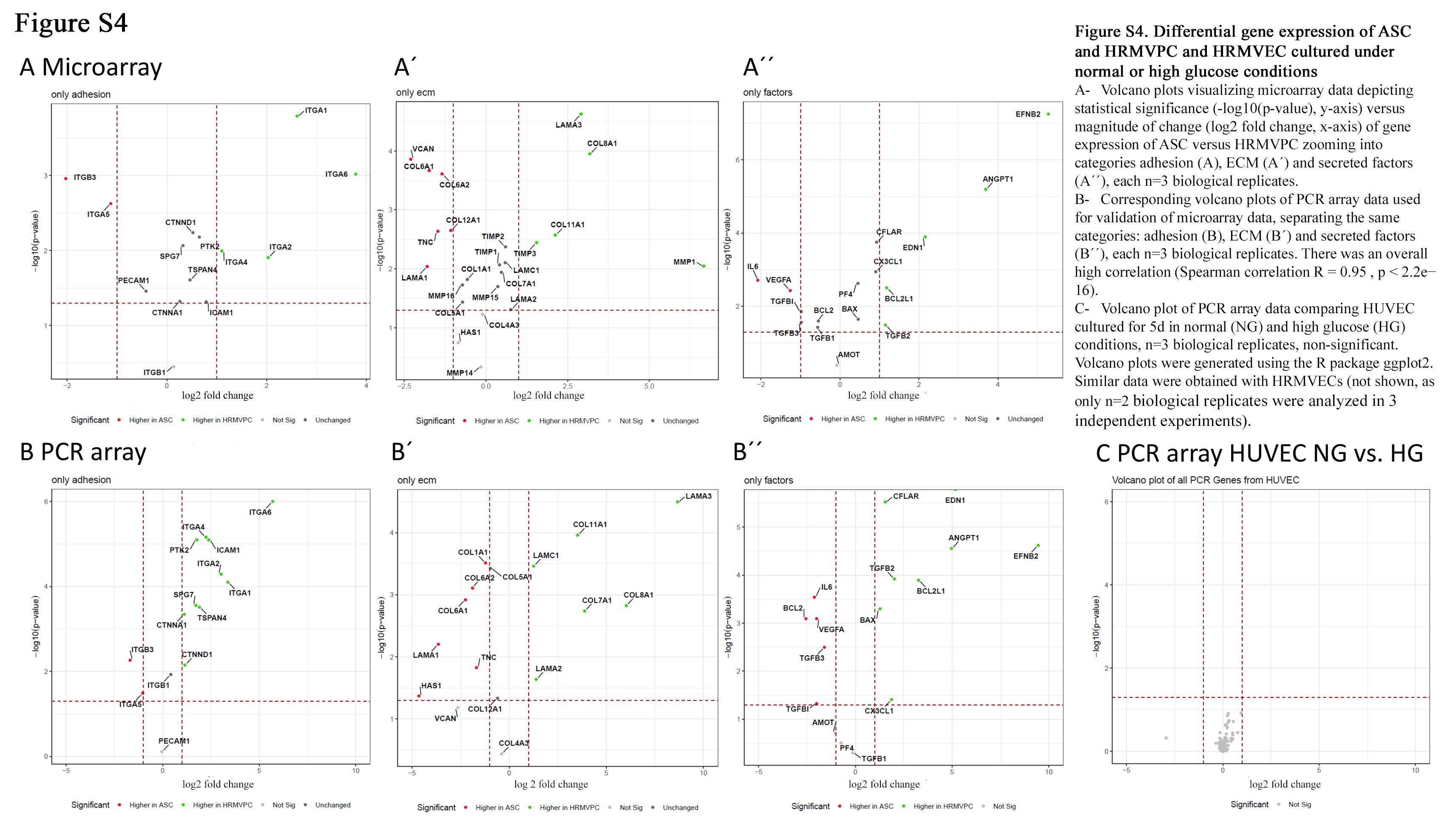

Supplement: Supplementary file 7 [file Image_5.TIF]
