# Supplementary material for: Pro-angiogenic Activity Discriminates Human Adipose-Derived Stromal Cells From Retinal Pericytes: Considerations for Cell-Based Therapy of Diabetic Retinopathy
Source: Front Cell Dev Biol. 2020 Jun 9;8:387. doi: 10.3389/fcell.2020.00387 (PMC7295949; doi:10.3389/fcell.2020.00387)
Supplement: TABLE S1 — Antibodies used for flow cytometry AF- Alexa Fluor, APC- Allophycocyanin, FITC- Fluorescein isothiocyanate, PE- Phycoerythrin. [file Table_1.pdf]

Supplementary Table 1

Antibodies used for flow cytometry

**Antibody**	**Clone**	**Company**
CD13-APC-Cy7	WM15	BioLegend
CD15-FITC	HI98	BD
CD29-Alexa Fluor 488	TS2/16	BioLegend
CD31-APC	WM59	eBioscience
CD34-PE	8G12	BD
CD44-APC	IM7	BioLegend
CD45-FITC	HI30	BD
CD49a-Alexa Fluor 647	TS2/7	AbD Serotec
CD49b-FITC	P1E6-C5	BioLegend
CD49d-PeCy7	9F10	BioLegend
CD49e-PE	NKI-SAM-1	BioLegend
CD49f-PE	GoH3	BioLegend
Integrin b7-APC	FIB504	BioLegend
CD73-PE	AD2	BD
CD90-APC	5E10	BD
CD105-APC	SN6	eBioscience
CD106-FITC	VCAM-1	BD
CD140a-PE	16A1	BioLegend
CD140b-APC	18A2	BioLegend
CD146-PE	TEA1/34	Beckman Coulter
CD202b-Alexa Fluor 488	33.1	BioLegend
CD248-Alexa Fluor 647	B1/35	BD
HLA-DR-FITC	L243	BioLegend
HLA-ABC-APC	REA230	Miltenyi
NG2-Alexa Fluor 488	9.2.27	EBioscience

AF- Alexa Fluor, APC- Allophycocyanin, FITC- Fluorescein isothiocyanate, PE- Phycoerythrin
