# Supplementary material for: Pro-angiogenic Activity Discriminates Human Adipose-Derived Stromal Cells From Retinal Pericytes: Considerations for Cell-Based Therapy of Diabetic Retinopathy
Source: Front Cell Dev Biol. 2020 Jun 9;8:387. doi: 10.3389/fcell.2020.00387 (PMC7295949; doi:10.3389/fcell.2020.00387)
Supplement: TABLE S2 — Gene list, Custom RT2 PCR Array. [file Table_2.pdf]

Table S2
Gene list, Custom RT2 PCR Array

PCR Array Catalog	CLAH31907

Position	Unigene	Refseq	Symbol	Description	Gname	RT2 Catalog
A01	Hs.500483	NM_001613	ACTA2	Actin, alpha 2, smooth muscle, aorta	AAT6/ACTSA/MYMY5	LPH31335A
A02	Hs.477887	NM_031850	AGTR1	Angiotensin II receptor, type 1	AG2S/AGTR1B/AT1/AT1AR/AT1B/AT1BR/AT1R/AT2R1/HAT1R	LPH35333A
A03	Hs.528051	NM_133265	AMOT	Angiomotin	-	LPH33105A
A04	Hs.369675	NM_001146	ANGPT1	Angiopoietin 1	AGP1/AGPT/ANG1	LPH36139A
A05	Hs.1239	NM_001150	ANPEP	Alanyl (membrane) aminopeptidase	APN/CD13/GP150/LAP1/P150/PEPN	LPH37785A
A06	Hs.744914	NM_178191	ATPIF1	ATPase inhibitory factor 1	ATPI/ATPIP/IP	
A07	Hs.624291	NM_004324	BAX	BCL2-associated X protein	BCL2L4	LPH29975A
A08	Hs.150749	NM_000633	BCL2	B-cell CLL/lymphoma 2	Bcl-2/PPP1R50	LPH38562A
A09	Hs.2399	NM_004995	MMP14	Matrix metallopeptidase 14 (membrane-inserted)	MMP-14/MMP-X1/MT-MMP/MT-MMP 1/MT1-MMP/MT1MMP/MTMMP1/WNCHRS	LPH36403A
A10	Hs.516966	NM_138578	BCL2L1	BCL2-like 1	BCL-XL/S/BCL2L/BCLX/BCLXL/BCLXS/Bcl-X/PPP1R52/bcl-xL/bcl-xS	
A11	Hs.57697	NM_001523	HAS1	Hyaluronan synthase 1	HAS	
A12	Hs.502302	NM_001752	CAT	Catalase	-	LPH32710A
B01	Hs.74034	NM_001753	CAV1	Caveolin 1, caveolae protein, 22kDa	BSCL3/CGL3/LCCNS/MSTP085/PPH3/VIP21	LPH31156A
B02	Hs.654379	NM_004357	CD151	CD151 molecule (Raph blood group)	GP27/MER2/PETA-3/RAPH/SFA1/TSPAN24	LPH39224A
B03	Hs.195727	NM_020404	CD248	CD248 molecule, endosialin	CD164L1/TEM1	
B04	Hs.54457	NM_004356	CD81	CD81 molecule	CVID6/S5.7/TAPA1/TSPAN28	LPH31622A
B05	Hs.390736	NM_003879	CFLAR	CASP8 and FADD-like apoptosis regulator	CASH/CASP8AP1/CLARP/Casper/FLAME/FLAME-1/FLAME1/FLIP/I-FLICE/MRIT/c-FLIP/c-FLIPL/c-FLIPR/c-FLIPS	LPH28513A
B06	Hs.523446	NM_080629	COL11A1	Collagen, type XI, alpha 1	CO11A1/COLL6/STL2	LPH29185A
B07	Hs.101302	NM_004370	COL12A1	Collagen, type XII, alpha 1	BA209D8.1/COL12A1L/DJ234P15.1	LPH31762A
B08	Hs.172928	NM_000088	COL1A1	Collagen, type I, alpha 1	EDSC/OI1/OI2/OI3/OI4	LPH31459A
B09	Hs.570065	NM_000091	COL4A3	Collagen, type IV, alpha 3 (Goodpasture antigen)	-	LPH38185A
B10	Hs.210283	NM_000093	COL5A1	Collagen, type V, alpha 1	EDSC	LPH33472A
B11	Hs.474053	NM_001848	COL6A1	Collagen, type VI, alpha 1	OPLL	LPH34986A
B12	Hs.420269	NM_001849	COL6A2	Collagen, type VI, alpha 2	PP3610	LPH34989A
C01	Hs.476218	NM_000094	COL7A1	Collagen, type VII, alpha 1	EBD1/EBDCT/EBR1/NDNC8	LPH31992A
C02	Hs.654548	NM_001850	COL8A1	Collagen, type VIII, alpha 1	C3orf7	LPH35318A

C03	Hs.513044	NM_001897	CSPG4	Chondroitin sulfate proteoglycan 4	HMW-MAA/MCSP/MCSPG/MEL-CSPG/MSK16/NG2	
C04	Hs.656653	NM_001903	CTNNA1	Catenin (cadherin-associated protein), alpha 1, 102kDa	CAP102	LPH29003A
C05	Hs.166011	NM_001331	CTNND1	Catenin (cadherin-associated protein), delta 1	CAS/CTNND/P120CAS/P120CTN/p120/p120(CAS)/p120(CTN)	LPH40910A
C06	Hs.531668	NM_002996	CX3CL1	Chemokine (C-X3-C motif) ligand 1	ABCD-3/C3Xkine/CXC3/CXC3C/NTN/NTT/SCYD1/fractalkine/neurotactin	
C07	Hs.594952	NM_001927	DES	Desmin	CSM1/CSM2/LGMD2R	LPH38968A
C08	Hs.511899	NM_001955	EDN1	Endothelin 1	ARCND3/ET1/HDLCQ7/PPET1/QME	
C09	Hs.183713	NM_001957	EDNRA	Endothelin receptor type A	ET-A/ETA/ETA-R/ETAR/ETRA/MFDA/hET-AR	LPH35904A
C10	Hs.149239	NM_004093	EFNB2	Ephrin-B2	EPLG5/HTKL/Htk-L/LERK5	
C11	Hs.446352	NM_004448	ERBB2	V-erb-b2 erythroblastic leukemia viral oncogene homolog 2, neuro/glioblastoma derived oncogene homolog (avian)	CD340/HER-2/HER-2/neu/HER2/MLN 19/NEU/NGL/TKR1	LPH34959A
C12	Hs.594454	NM_002019	FLT1	Fms-related tyrosine kinase 1 (vascular endothelial growth factor/vascular permeability factor receptor)	FLT/FLT-1/VEGFR-1/VEGFR1	
D01	Hs.643447	NM_000201	ICAM1	Intercellular adhesion molecule 1	BB2/CD54/P3.58	LPH30104A
D02	Hs.634632	NM_005534	IFNGR2	Interferon gamma receptor 2 (interferon gamma transducer 1)	AF-1/IFGR2/IFNGT1/IMD28	
D03	Hs.654458	NM_000600	IL6	Interleukin 6 (interferon, beta 2)	BSF2/HGF/HSF/IFNB2/IL-6	LPH34215A
D04	Hs.644352	NM_181501	ITGA1	Integrin, alpha 1	CD49a/VLA1	LPH41417A
D05	Hs.482077	NM_002203	ITGA2	Integrin, alpha 2 (CD49B, alpha 2 subunit of VLA-2 receptor)	BR/CD49B/GPIa/HPA-5/VLA-2/VLAA2	LPH37316A
D06	Hs.440955	NM_000885	ITGA4	Integrin, alpha 4 (antigen CD49D, alpha 4 subunit of VLA-4 receptor)	CD49D/IA4	LPH32094A
D07	Hs.505654	NM_002205	ITGA5	Integrin, alpha 5 (fibronectin receptor, alpha polypeptide)	CD49e/FNRA/VLA-5/VLA5A	LPH36822A
D08	Hs.133397	NM_000210	ITGA6	Integrin, alpha 6	CD49f/ITGA6B/VLA-6	LPH30151A
D09	Hs.643813	NM_002211	ITGB1	Integrin, beta 1 (fibronectin receptor, beta polypeptide, antigen CD29 includes MDF2, MSK12)	CD29/FNRB/GPIIA/MDF2/MSK12/VLA-BETA/VLAB	LPH35794A
D10	Hs.218040	NM_000212	ITGB3	Integrin, beta 3 (platelet glycoprotein IIIa, antigen CD61)	BDPLT16/BDPLT2/CD61/GP3A/GPIIIa/GT	
D11	Hs.224012	NM_000214	JAG1	Jagged 1	AGS/AHD/AWS/CD339/HJ1/JAGL1	

D12	Hs.479754	NM_000222	KIT	V-kit Hardy-Zuckerman 4 feline sarcoma viral oncogene homolog	C-Kit/CD117/PBT/SCFR	LPH36416A
E01	Hs.270364	NM_005559	LAMA1	Laminin, alpha 1	LAMA/PTBHS/S-LAM-alpha	LPH30627A
E02	Hs.200841	NM_000426	LAMA2	Laminin, alpha 2	LAMM	LPH40566A
E03	Hs.436367	NM_000227	LAMA3	Laminin, alpha 3	BM600/E170/LAMNA/LOCS	LPH29094A
E04	Hs.609663	NM_002293	LAMC1	Laminin, gamma 1 (formerly LAMB2)	LAMB2	LPH34148A
E05	Hs.23581	NM_002303	LEPR	Leptin receptor	CD295/LEP-R/LEPRD/OB-R/OBR	LPH41387A
E06	Hs.599039	NM_006500	MCAM	Melanoma cell adhesion molecule	CD146/MUC18	LPH29661A
E07	Hs.83169	NM_002421	MMP1	Matrix metallopeptidase 1 (interstitial collagenase)	CLG/CLGN	
E08	Hs.80343	NM_002428	MMP15	Matrix metallopeptidase 15 (membrane-inserted)	MT2-MMP/MTMMP2/SMCP-2	
E09	Hs.546267	NM_005941	MMP16	Matrix metallopeptidase 16 (membrane-inserted)	C8orf57/MMP-X2/MT-MMP2/MT-MMP3/MT3-MMP	LPH36293A
E10	Hs.495473	NM_017617	NOTCH1	Notch 1	AOS5/AOVD1/TAN1/hN1	LPH35648A
E11	Hs.487360	NM_024408	NOTCH2	Notch 2	AGS2/HJCYS/hN2	LPH33915A
E12	Hs.8546	NM_000435	NOTCH3	Notch 3	CADASIL/CASIL/IMF2/LMNS	LPH29568A
F01	Hs.131704	NM_003873	NRP1	Neuropilin 1	BDCA4/CD304/NP1/NRP/VEGF165R	LPH30269A
F02	Hs.471200	NM_003872	NRP2	Neuropilin 2	NP2/NPN2/PRO2714/VEGF165R2	LPH32427A
F03	Hs.74615	NM_006206	PDGFRA	Platelet-derived growth factor receptor, alpha polypeptide	CD140A/PDGFR-2/PDGFR2/RHEPDGFRA	LPH33997A
F04	Hs.509067	NM_002609	PDGFRB	Platelet-derived growth factor receptor, beta polypeptide	CD140B/IBGC4/IMF1/JTK12/PDGFR/PDGFR-1/PDGFR1	LPH31954A
F05	Hs.514412	NM_000442	PECAM1	Platelet/endothelial cell adhesion molecule	CD31/CD31/EndoCAM/GPIIA'/PECA1/PECAM-1/endoCAM	
F06	Hs.81564	NM_002619	PF4	Platelet factor 4	CXCL4/PF-4/SCYB4	
F07	Hs.491582	NM_000930	PLAT	Plasminogen activator, tissue	T-PA/TPA	LPH30896A
F08	Hs.77274	NM_002658	PLAU	Plasminogen activator, urokinase	ATF/BDPLT5/QPD/UPA/URK/u-PA	LPH32806A
F09	Hs.647450	NM_006404	PROCR	Protein C receptor, endothelial	CCCA/CCD41/EPCR	
F10	Hs.201978	NM_000962	PTGS1	Prostaglandin-endoperoxide synthase 1 (prostaglandin G/H synthase and cyclooxygenase)	COX1/COX3/PCOX1/PES-1/PGG/HS/PGHS-1/PGHS1/PHS1/PTGHS	
F11	Hs.395482	NM_005607	PTK2	PTK2 protein tyrosine kinase 2	FADK/FAK/FAK1/FRNK/PPP1R71/p125FAK/pp125FAK	LPH38244A
F12	Hs.24950	NM_003617	RGS5	Regulator of G-protein signaling 5	MST092/MST106/MST129/MSTP032/MSTP092/MSTP106/MSTP129	LPH35095A
G01	Hs.185597	NM_003119	SPG7	Spastic paraplegia 7 (pure and complicated autosomal recessive)	CAR/CMAR/PGN/SPG5C	LPH40791A
G02	Hs.68061	NM_021972	SPHK1	Sphingosine kinase 1	SPHK	LPH39086A
G03	Hs.89640	NM_000459	TEK	TEK tyrosine kinase, endothelial	CD202B/TIE-2/TIE2/VMCM/VMCM1	

G04	Hs.645227	NM_000660	TGFB1	Transforming growth factor, beta 1	CED/DPD1/LAP/TGFB/TGFbeta	LPH31036A
G05	Hs.133379	NM_003238	TGFB2	Transforming growth factor, beta 2	LDS4/TGF-beta2	LPH30205A
G06	Hs.713281	NM_003239	TGFB3	Transforming growth factor, beta 3	ARVD/ARVD1/RNHF/TGF-beta3	LPH32535A
G07	Hs.369397	NM_000358	TGFBI	Transforming growth factor, beta-induced, 68kDa	BIGH3/CDB1/CDG2/CDGG1/CSD/CSD1/CSD2/CSD3/EBMD/LCD1	LPH32632A
G08	Hs.522632	NM_003254	TIMP1	TIMP metallopeptidase inhibitor 1	CLGI/EPA/EPO/HCI/TIMP	
G09	Hs.633514	NM_003255	TIMP2	TIMP metallopeptidase inhibitor 2	CSC-21K/DDC8	
G10	Hs.644633	NM_000362	TIMP3	TIMP metallopeptidase inhibitor 3	HSMRK222/K222/K222TA2/SFD	
G11	Hs.143250	NM_002160	TNC	Tenascin C	150-225/DFNA56/GMEM/GP/HXB/JI/TN/TN-C	LPH28983A
G12	Hs.654836	NM_003271	TSPAN4	Tetraspanin 4	NAG-2/NAG2/TETRASPAN/TM4SF7/TSPAN-4	LPH41429A
H01	Hs.643801	NM_004385	VCAN	Versican	CSPG2/ERVR/GHAP/PG-M/WGN/WGN1	LPH28958A
H02	Hs.73793	NM_003376	VEGFA	Vascular endothelial growth factor A	MVCD1/VEGF/VPF	LPH31855A
H03	Hs.520640	NM_001101	ACTB	Actin, beta	BRWS1/PS1TP5BP1	LPH28471A
H04	Hs.534255	NM_004048	B2M	Beta-2-microglobulin	-	LPH28472A
H05	Hs.592355	NM_002046	GAPDH	Glyceraldehyde-3-phosphate dehydrogenase	G3PD/GAPD/HEL-S-162eP	LPH31725A
H06	Hs.546285	NM_001002	RPLP0	Ribosomal protein, large, P0	L10E/LP0/P0/PRLP0/RPP0	LPH28473A
H07	Hs.412707	NM_000194	HPRT1	Hypoxanthine phosphoribosyltransferase 1	HGPRT/HPRT	LPH37575A
H08	N/A	N/A	RTC	N/A	N/A	
H09	N/A	N/A	RTC	N/A	N/A	
H10	N/A	N/A	PPC	N/A	N/A	
H11	N/A	N/A	PPC	N/A	N/A	
H12	N/A	N/A	GDC	N/A	N/A	
